# Osmium Nanoparticles-Polypropylene Hollow Fiber Membranes Applied in Redox Processes

**DOI:** 10.3390/nano11102526

**Published:** 2021-09-27

**Authors:** Gheorghe Nechifor, Florentina Mihaela Păncescu, Alexandra Raluca Grosu, Paul Constantin Albu, Ovidiu Oprea, Szidonia-Katalin Tanczos, Constantin Bungău, Vlad-Alexandru Grosu, Andreia Pîrțac, Aurelia Cristina Nechifor

**Affiliations:** 1 Department of Analytical Chemistry and Environmental Engineering, University Politehnica of Bucharest, 011061 Bucharest, Romania; ghnechifor@gmail.com (G.N.); florynicorici@yahoo.com (F.M.P.); alexandra.raluca.miron@gmail.com (A.R.G.); andreia.pascu@yahoo.ro (A.P.); aureliacristinanechifor@gmail.com (A.C.N.); 2 National Institute for Research and Development in Physics and Nuclear Engineering, Radioisotopes and Radiation Metrology Department, 023465 Măgurele, Romania; 3 Department of Inorganic Chemistry, Physical Chemistry and Electrochemistry, University Politehnica of Bucharest, 011061 Bucharest, Romania; ovidiu.oprea@upb.ro; 4 Department of Bioengineering, Sapientia Hungarian University of Transylvania, 500104 Miercurea-Ciuc, Romania; tczszidonia@yahoo.com; 5 Department of Engineering and Management, Faculty of Management and Technological Engineering, University of Oradea, 410087 Oradea, Romania; bungau@gmail.com; 6 Department of Electronic Technology and Reliability, Faculty of Electronics, Telecommunications and Information Technology, University Politehnica of Bucharest, 061071 Bucharest, Romania

**Keywords:** metal nanoparticles-polymer membranes, osmium nanoparticle, osmium nanoparticles-polymer membranes, polypropylene hollow fiber membranes, osmium nanoparticles-polypropylene hollow fiber membranes, redox processes, membrane reactor, reduction, oxidation, *p-*nitrophenol, 10-undecylenic acid

## Abstract

Composite membranes play a very important role in the separation, concentration, and purification processes, but especially in membrane reactors and membrane bioreactors. The development of composite membranes has gained momentum especially through the involvement of various nanoparticles, polymeric, oxide, or metal, that have contributed to increasing their reactivity and selectivity. This paper presents the preparation and characterization of an active metal nanoparticle-support polymer type composite membrane, based on osmium nanoparticles obtained in situ on a polypropylene hollow fiber membrane. Osmium nanoparticles are generated from a solution of osmium tetroxide in *tert-*butyl alcohol by reduction with molecular hydrogen in a contactor with a polypropylene membrane. The composite osmium-polypropylene hollow fiber obtained membranes (Os-PPM) were characterized from the morphological and structural points of view: scanning electron microscopy (SEM), high resolution SEM (HR-SEM), energy dispersive spectroscopy analysis (EDAX), X-ray diffraction analysis (XRD), Fourier transform Infrared (FTIR) spectroscopy, thermal gravimetric analysis, and differential scanning calorimetry (TGA, DSC). The process performance was tested in a redox process of *p-*nitrophenol and 10-undecylenic (10-undecenoic) acid, as a target substance of biological or biomedical interest, in solutions of lower aliphatic alcohols in a membrane contactor with a prepared composite membrane. The characteristics of osmium nanoparticles-polypropylene hollow fiber membranes open the way to biological and biotechnological applications. These membranes do not contaminate the working environment, operate at relatively low temperatures, provide a large contact area between reactants, allow successive oxidation and reduction operations in the same module, and help to recover the reaction mass by ultrafiltration. The results obtained show that the osmium-polypropylene composite membrane allows the reduction of *p-*nitrophenol or the oxidation of 10-undecylenic acid, the conversion depending on the concentration in the lower aliphatic alcohol, the nature of the lower aliphatic alcohol, and the oxidant or reducing flow through the membrane contactor.

## 1. Introduction

**Figure 1:**
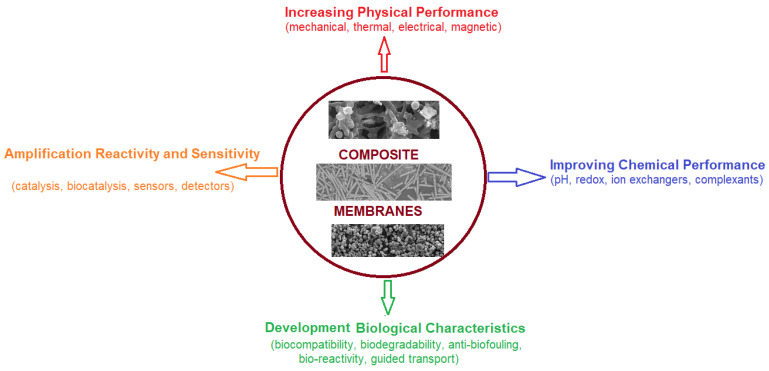
Composite membranes based on nanoparticles and the specific effects.

The development of membranes and membrane processes involving composite membranes has gained great importance with the progress made in obtaining nanoparticles with a well-controlled composition, shape, and size distribution [1,2]. Obviously, the nature of the nanoparticles used in obtaining membranes is of overwhelming importance for selectivity, reactivity, or effect on membrane processes (Figure 1). Thus, polymeric nanoparticles based on carbon, oxide, metals, or their compounds have been used for the preparation of composite membranes both in separation processes and in various reaction processes: esterification, etherification, reduction, and oxidation [2,3,4]. At the same time, intense studies have followed the influence of the size and shape of nanoparticles from a certain material on the performance of composite membranes [5,6]. The composite membranes made using metal nanoparticles (silver, gold, copper, nickel, or palladium) have a special place, which brought significant improvements in selectivity and reactivity [7,8,9,10].

As outlined in Figure 1, the use of nanoparticles in the realization of composite membranes and the processes based on them has brought important benefits to users, such as:

- Increasing physical performance: mechanical, thermal, electrical, or magnetic [11,12,13,14],
- Improving chemical performance: pH, redox, ion exchange, complexation [15,16,17,18],
- Amplification of activity and sensitivity: catalysis, bio-catalysis, sensors, detector [19,20,21,22], and
- Development of biological characteristics: biocompatibility, biodegradability, anti-biofouling, guided transport [22,23,24,25,26].

Composite membranes based on metal nanoparticles fit in a very special way in all these directions of development of membrane processes. However, the reactivity or catalytic activity are among the best studied and used characteristics of metal nanoparticle–polymer based composite membranes [27,28,29,30,31]. Although they have special properties, osmium and its compounds have been used relatively little in membrane processes [32,33], the main application being the metallization of polymeric or biological membranes for studies in electron microscopy [34].

Due to its chemical inertia, even against aggressive environments [35], osmium can be an interesting membrane material for the construction of membrane reactors aimed at hydrogenation or oxidation [30,36].

Making metal-polymer composite membranes is of interest from the perspective of increasing the physical-chemical and process performances, especially when it is represented by a chemical reaction. The use of osmium as a membrane material can be an economic disadvantage due to the high price, but when used as nanoparticles generated from an accessible material, such as osmium tetroxide, this aspect is overcome (Figures S1–S4).

At the same time, osmium obtained by reducing osmium tetroxide waste on a porous polymeric support can be a useful means of recovery, especially since in catalytic reactions, it is known for selectivity [37,38], regiospecificity [39,40], and stereospecificity [41].

The osmium-polymer (Os-P) membranes can be used in reduction or oxidation reactions, and as target substances can be considered those of interest for environmental, biological, or biomedical protection, such as *p*-nitrophenol [42,43,44,45,46] and 10-undecylenic acid [47,48,49,50,51].

The characteristics of osmium nanoparticles-polypropylene hollow fiber membranes open the path to biological and biotechnological applications. We can mention that these membranes:

- Do not contaminate the working environment,
- Operate at relatively low temperatures,
- Provide a large contact area between reactants,
- Help to recover the reaction mass by ultrafiltration, and
- Allow successive oxidation and reduction operations in the same module.

This paper presents the preparation and characterization of a composite membrane of active metal nanoparticle-support polymer type, based on osmium nanoparticles obtained in situ on a polypropylene hollow fiber membrane and its use in the transformation of 10-undecylenic acid or *p-*nitrophenol by oxidation and reduction.

## 2. Experiments

### 2.1. Materials

The reagents used in the presented work were of analytical grade and were used without other purification.

Methanol, ethanol, *i-*propanol and *tert-*butanol were purchased from Merck (Merck KGaA, Darmstadt, Germany). Osmium tetroxide (OsO_4_), *p-*nitrophenol, and 10-undecylenic (10-undecenoic) acid (>95%) were purchased from Sigma-Aldrich (Merck KGaA, Darmstadt, Germany).

The purified water characterized by 18.2 μS/cm conductivity was obtained with a RO Millipore system (MilliQ® Direct 8 RO Water Purification System, Merck KGaA, Darmstadt, Germany).

**Figure 2:**
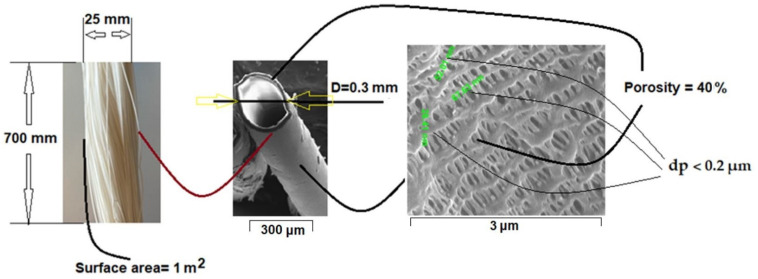
The typical characteristics of the polypropylene hollow fiber membrane (PPM).

The hollow polypropylene fibers used as membrane support (PPM) were provided by GOST Ltd., Perugia, Italy (Figure 2) [52,53].

Polypropylene hollow fiber membranes at a length of 700 mm and a diameter of 0.3 mm are assembled so as to achieve a filtering surface of 1 m^2^ [52,54]. The fibers have a porosity of 40% [54,55] and a pore diameter of less than 0.2 µm [56] (Figure 2). This type of module with polypropylene hollow fiber membranes allows operation over the entire pH range, in oxidizing or reducing solutions at temperatures up to 100 °C without dimensional changes and can be sterilized by known physical-chemical methods [57,58,59].

### 2.2. Procedures

#### 2.2.1. Preparation of Osmium Nanoparticles on Polypropylene Hollow Fiber Membranes

**Figure 3:**
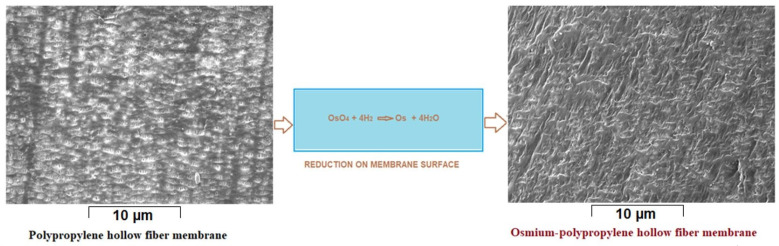
Obtaining osmium nanoparticles on polypropylene hollow fiber membrane.

To make osmium nanoparticles-polypropylene hollow fibers composite membranes, on the support membrane is deposited metallic osmium from the reaction of osmium tetroxide dissolved in *tert-*butyl alcohol (1.5 and 2.0 g OsO_4_ dissolved in 2500 mL *tert-*butyl alcohol), at a temperature of 25 °C (Figure 3), when the chemical reaction takes place according to Equation (1).

OsO_4_ + 4 H_2_ → Os + 4 H_2_O (1)

The stages of reduction of osmium tetroxide on the polypropylene membrane are:

- Formation of the osmium tetroxide solution by dissolving 1 g of solid substance in 1000 mL of *tert-*butyl alcohol,
- Immersion of the fiber bundle in the alcoholic solution of osmium tetroxide,
- Reduction of osmium tetroxide with molecular hydrogen,
- Ethanol washing of osmium nanoparticles-polypropylene hollow fibers composite membranes, and
- Drying in a vacuum oven (60 °C).

The nanocomposite membrane samples containing osmium were characterized by scanning electron microscopy (SEM) and high resolution scanning electron microscopy (HR-SEM), energy dispersive spectroscopy analysis (EDAX), X-ray diffraction analysis (XRD), atomic absorption spectroscopy, and thermal analysis (TG, DSC).

For X-ray diffraction analysis (XRD), atomic absorption spectroscopy, and thermal analysis (TG, DSC) the samples were ground at a colloidal ball mill (Retsch PM 100, VIOLA—Shimadzu, Bucharest, Romania) provided with ceramic grinding bodies.

#### 2.2.2. Carrying out the Process of Oxidation or Reduction

**Figure 4:**
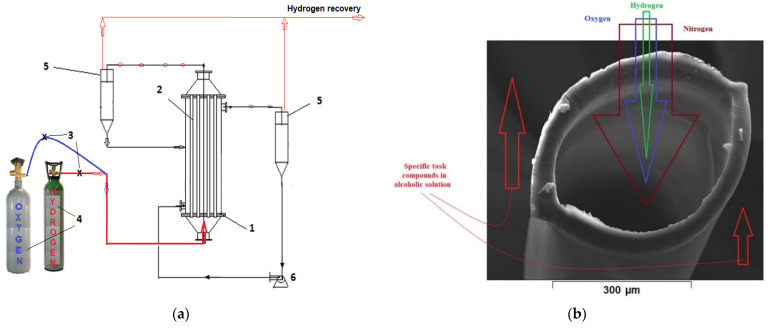
(**a**) The schematic presentation of the 10-undecylenic acid oxidation or *p-*nitrophenol reduction installation: 1: membrane contactor; 2: polypropylene hollow fiber membranes; 3, 4: hydrogen or oxygen source; 5: gas-liquid separator; 6: pump for solution in *tert-*butyl alcohol (methanol, ethanol, or propanol); and (**b**) fluid circulation through the system.

The impregnated membrane bundle was mounted in a reaction module similar to that extensively described in our previous works [60,61,62]. A bundle of composite fibers was inserted into this housing and subsequently immobilized with acrylic polymer, thus obtaining a membrane contactor similar to a tubular heat exchanger. This module was placed in the working installation (Figure 4a). The circulation of fluids through a membrane is shown in Figure 4b.

The solution of *p-*nitrophenol or 10-undecylenic acid in *tert-*butyl alcohol (ethanol or propanol) was conveyed through the membranes, and hydrogen or molecular oxygen was transported at normal pressure (1 atm) through the composite membranes.

Three samples of 1 mL from the alcoholic *p-*nitrophenol or 10-undecylenic acid synthesized solutions were periodically spectrophotometrically analyzed [62,63].

The conversion (*η* %) of analytes calculated using the solution’s concentration (2) or absorbance (3) [64,65] were:

(2) $$\eta \left(\%\right)=\frac{\left(c_{0}-c_{f}\right)}{c_{0}}\times 100$$

where *c_f_* is the final concentration of the solute (10-undecylenic acid or *p-*nitrophenol) and *c*_o_ is the initial concentration of solute (10-undecylenic acid or *p-*nitrophenol).

(3) $$\eta \left(\%\right)=\frac{\left(A_{0}-A_{s}\right)\;}{A_{0}}\;\times 100$$

*A*_0_ is the initial sample solution absorbance and *A_s_* is the current sample absorbance.

If the reduction of *p-*nitrophenol leads to *p-*aminophenol, the oxidation of 10-undecylenic acid leads to a variety of reaction products. That is why in this study, only the conversion was followed. However, to illustrate the complexity of the reaction mass, 10 mL of solutes was subjected to chromatography on a 60 µm silica column and the separated compounds were identified through Fourier transform infrared (FTIR) spectrometry (see Table S1).

After the processing, nitrogen was introduced into the module for two hours to remove hydrogen or oxygen. In the specific case of oxidation, after the process, nitrogen was introduced into the module for two hours to remove oxygen, then hydrogen for two hours to reduce any traces of osmium oxide, and, finally, nitrogen was introduced again for two hours. The module was opened for membrane sampling for analysis after the reaction mass was aspirated, through membranes (a vacuum nanofiltration was performed at 100 mm H_2_O to maximize the reaction mass (permeate) and the retention of nanoparticles and nanoparticle aggregates of osmium (concentrate)).

Successive nitrogen washing and hydrogen gas reduction operations ensured an osmium-free reaction mass and recovery of any osmium loss. The operations described are also mandatory to ensure the safety at work of the laboratory and the staff.

### 2.3. Equipment

The microscopy studies, SEM and HR-SEM, were performed on a Hitachi S4500 system (Hitachi High—Technologies Europe GmbH, Mannheim, Germany).

X-ray diffraction analyses (XRD) were recorded using PANalytical X’Pert Pro MPD equipment (PANalytical B.V., Almelo, The Netherlands) with a CuKαCradiation source, and 2θ measurement range, from 10 to 90 °C.

Thermal analysis (TG, DSC) was performed with a STA 449C Jupiter apparatus, from Netzsch (NETZSCH-Gerätebau GmbH, Selb, Germany). Each sample weighed approximately 10 mg. The samples were placed in an open alumina crucible and heated up to 900 °C with 10 K∙min^−1^ rate, under flow of 50 mL∙min^−1^ dried air. As reference, we used an empty alumina crucible. The evolved gases were analyzed with a FTIR Tensor 27 from Bruker (Bruker Co., Ettlingen, Germany), equipped with a thermostat gas cell.

The UV–Vis analyses of the 10-undecylenic acid solutions were done on a Spectrophotometer CamSpec M550 (Spectronic CamSpec Ltd., Leeds, UK).

The electrochemical processes were followed up with a PARSTAT 2273 Potentiostat (Princeton Applied Research, AMETEK Inc., Berwyn, PA, USA). It has been used a glass cell with three electrodes setup.

Cyclic voltammetry was performed for a potential sweep between −0.5 V and +1.23 V, at a scan rate of 50 mV/s. The experimental procedure took place at room temperature.

The UV–Vis studies on the nanoparticles samples composition were performed on dual-beam UV equipment—Varian Cary 50 (Agilent Technologies Inc., Santa Clara, CA, USA)—at a resolution of 1 nm, spectral bandwidth 1.5 nm, and 300 nm/s scan rate. The samples’ UV–Vis spectra were recorded for a wavelength from 200 nm to 800 nm, at room temperature, using 10 mm quartz cells.

The pH of the medium was followed up with a combined selective electrode (HI 4107, Hanna Instruments Ltd, Leighton Buzzard, UK) and a multi-parameter system (HI 5522, Hanna Instruments Ltd., Leighton Buzzard, UK).

To assess and validate the content in metal ions, the atomic absorption spectrometer AAnalyst 400 AA Spectrometer (Perkin Elmer Inc., Shelton, CT, USA) with a single-element hollow-cathode lamp was used, driven by WinLab32—AA software (Perkin Elmer Inc., Shelton, CT, USA).

## 3. Results and Discussions

The present study used osmium nanoparticles-polypropylene hollow fiber membranes selected from previous experiments on osmium nanoparticles-polymer membranes [63], which included three polymers of different hydrophobicity: polyethylene, polysulfone, and cellulose acetate.

The choice of polypropylene hollow fiber membrane was determined both by the nature of the environment that the studies in this paper address (alcoholic solutions) and by the need for a large contact area.

Polypropylene hollow fiber membrane has the advantage of a chemical resistance over the entire pH range, but also in oxidizing, reducing, or temperature environments up to 90–100 °C.

This paper presents the recovery of osmium tetroxide, left from various stages of processing in electron microscopy, as follows:

- The preparation of composite membranes based on osmium-polypropylene hollow fiber (Os-PP), by in situ reduction of the osmium tetroxide from *tert-*butyl alcohol solution with molecular hydrogen,
- Morpho-structural characterization of the obtained osmium-polypropylene hollow fiber composite membranes,
- The reduction of *p-*nitrophenol with molecular hydrogen in a module using osmium-polypropylene hollow fiber composite membranes, and
- Oxidation of 10-undecylenic acid with osmium-polypropylene hollow fiber composite membranes using molecular oxygen.

### 3.1. The Preparation and Characterization of the Composite Membrane Osmium-Polypropylene Hollow Fiber (Os-PP) by In Situ Reduction of Osmium Tetroxide

Obtaining a composite membrane intended for a membrane reactor must combine the special physical-chemical resistance of the support with the selectivity and versatility of metallic nanoparticles.

The membrane support chosen for the osmium nanoparticles-polymer composite membrane is the polypropylene hollow fiber membrane that ensures the possibility of working in aqueous or non-aqueous environments, over the entire pH range, at temperatures up to 90–100 °C and multiple washing or sterilization variants (hypochlorite, hydrogen peroxide, alkaline, or basic solutions).

**Figure 5:**
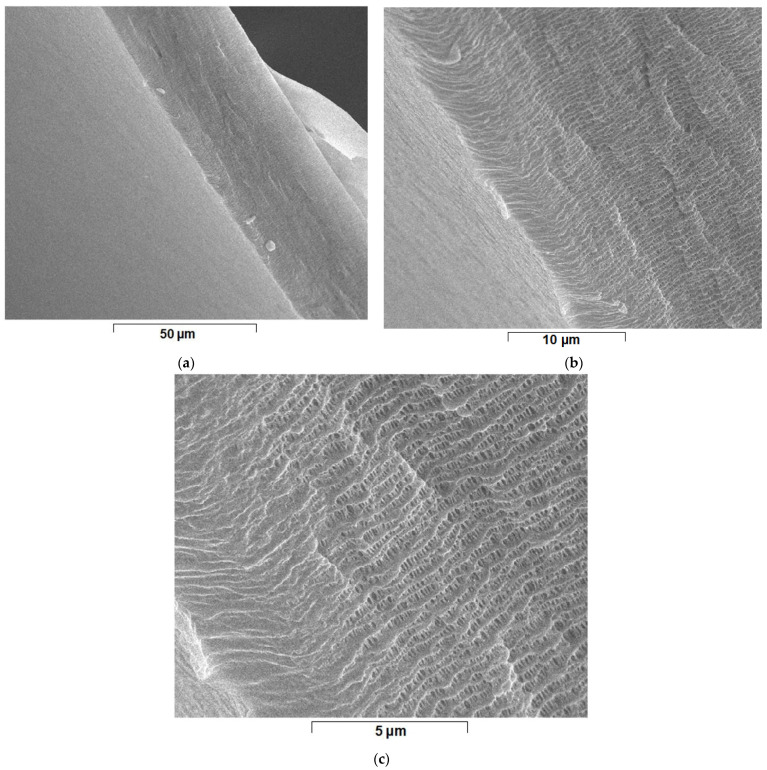
Scanning electron microscopy (SEM) of the uniformity of porosity and dimensional distribution of polypropylene hollow fibers membranes pores at various magnitudes: (**a**) ×2000; (**b**) ×8000; and (**c**) ×20,000.

**Figure 6:**
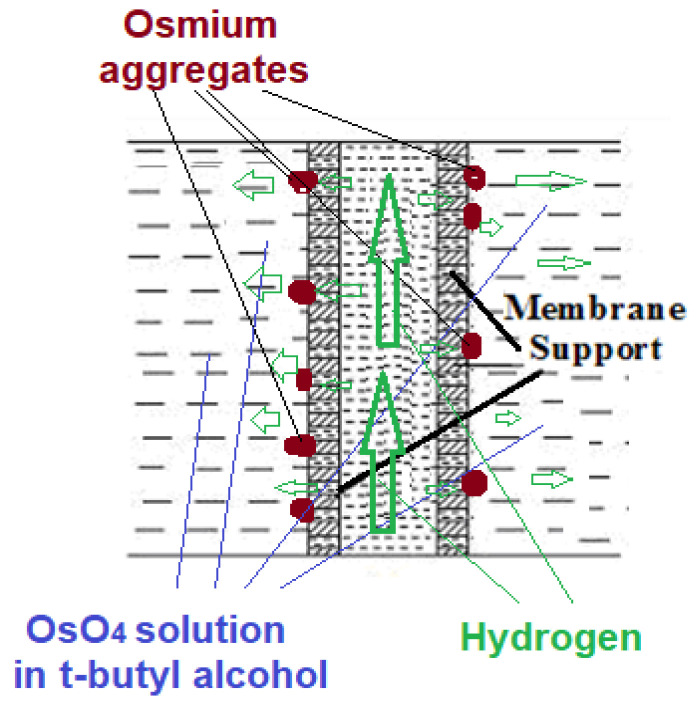
Schematic presentation of the contactor for reducing osmium tetroxide dissolved in *tert-*butyl alcohol at aggregates of metallic osmium nanoparticle with molecular hydrogen.

**Figure 7:**
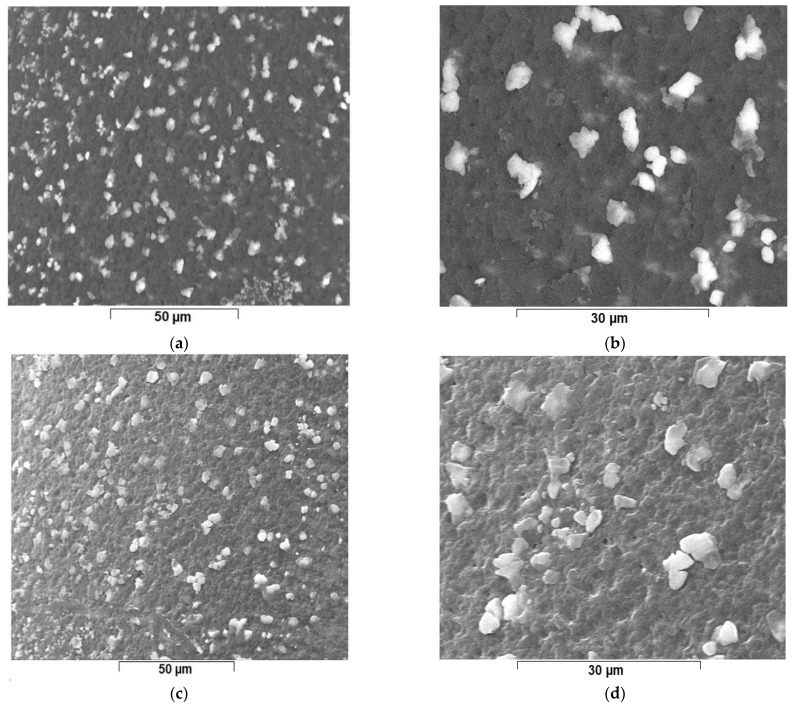
The scanning electron microscopy of the membrane support in the pores of which nanoparticle aggregates of metallic osmium have developed: raw osmium nanoparticles-polypropylene hollow fibers composite membrane 1 (Os-PP1) (**a**) ×2000 and (**b**) ×5000; and raw osmium nanoparticles-polypropylene hollow fibers composite membrane 2 (Os-PP2) (**c**) ×2000 and (**d**) ×5000.

In this study, the most important characteristics were the porosity (minimum 40%), pore diameter (less than 0.2 µm), and small thickness of approx. 20 µm (Figure 5 and Figure S4). These characteristics allowed the intimate contact of hydrogen, through the membrane contactor which was also the membrane preparation installation (Figure 4), with the solution of osmium tetroxides in *tert-*butyl alcohol, in order to reduce the metallic osmium that thus develops in the pores (Figure 6 and Figure 7). Molecular hydrogen was conveyed through the polypropylene hollow fibers membranes at a flow rate of 1 L/min, and the solution of osmium tetroxide in *tert-*butyl was maintained outside the fibers.

The concentration of the solution of osmium tetroxide in *tert-*butyl alcohol was chosen at 0.6 g/L for the Os-PP1 membrane and 0.8 g/L for the Os-PP2 membrane. The concentrations of the solutions were chosen in order to be able to compare these composite membranes with the previously obtained and reported membrane, Os-PP0 [64]. The concentration of the solution of osmium tetroxide in *tert-*butyl alcohol was 0.4 g/L. The increase in concentration of osmium tetroxide had in view obtaining a high concentration of osmium atoms at the surface of the raw membranes (Figure 7) highlighted by energy dispersive spectroscopy analysis (EDAX) (Table 1).

Although the local analyses (on the surface) showed the presence of osmium nanoparticles and nanoparticles aggregates, the X-ray diffraction analysis (XRD) being a global method (a quantity of Os-PP membranes ground at the mill was analyzed) was more difficult to interpret, but the lines specific to metallic osmium were still highlighted (see Figure S5 in the Supplementary Material).

**Figure 8:**
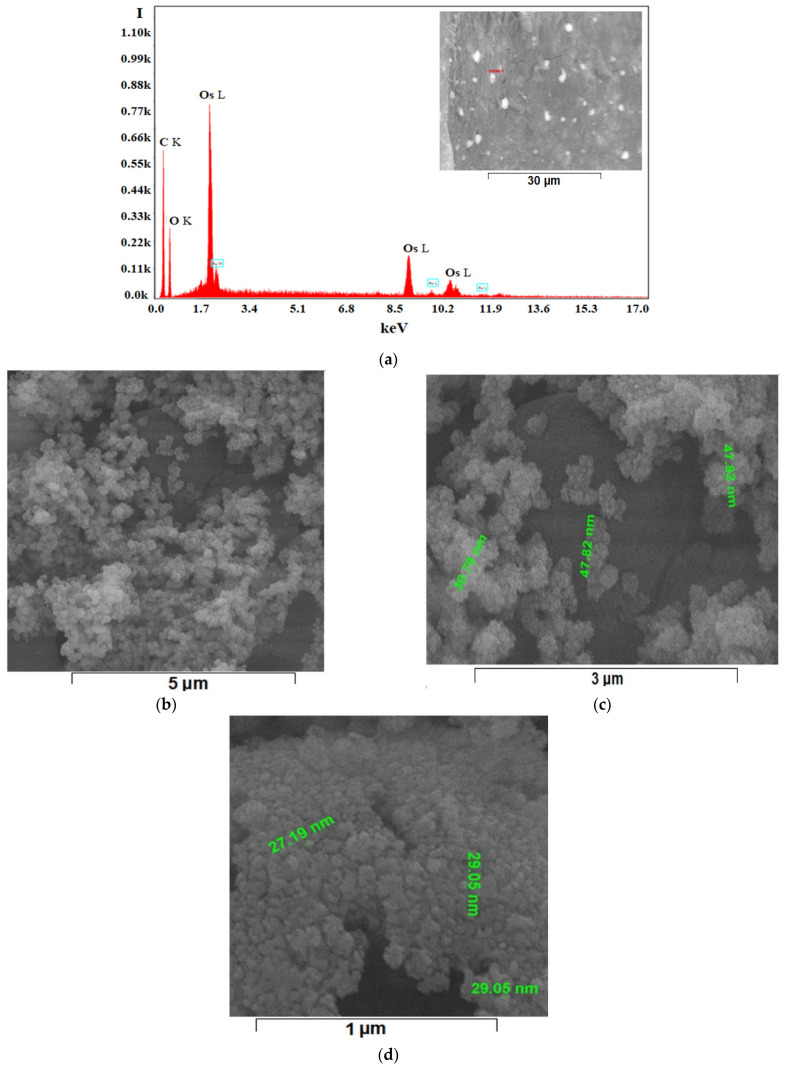
Formal energy dispersive spectroscopy analysis (EDAX) for the raw osmium nanoparticles-polypropylene hollow fibers composite membrane (Os-PP) (**a**), and high-resolution scanning electron microscopy (HR-SEM) of the specific osmium nanoparticles distribution on raw membrane surface: ×20,000 (**b**); ×50,000 (**c**); and ×100,000 (**d**).

To overcome this aspect, extensive energy dispersive spectroscopy analysis (EDAX) analyses were performed. The EDAX diagrams, having a formal aspect presented in Figure 8a and Figure S6, showed the presence of carbon and osmium on the surface, but also of oxygen, which comes both from the conditioning agent of polypropylene hollow fibers membranes and from the working solvent, or the hydrating water that appears in the reduction of osmium tetroxide (see also Equation (1)).

The dimensions of osmium nanoparticles (Figure 8b–d) were tracked by detailing the nanoparticle aggregates on the raw osmium nanoparticles-polypropylene hollow fibers composite membranes.

**Figure 9:**
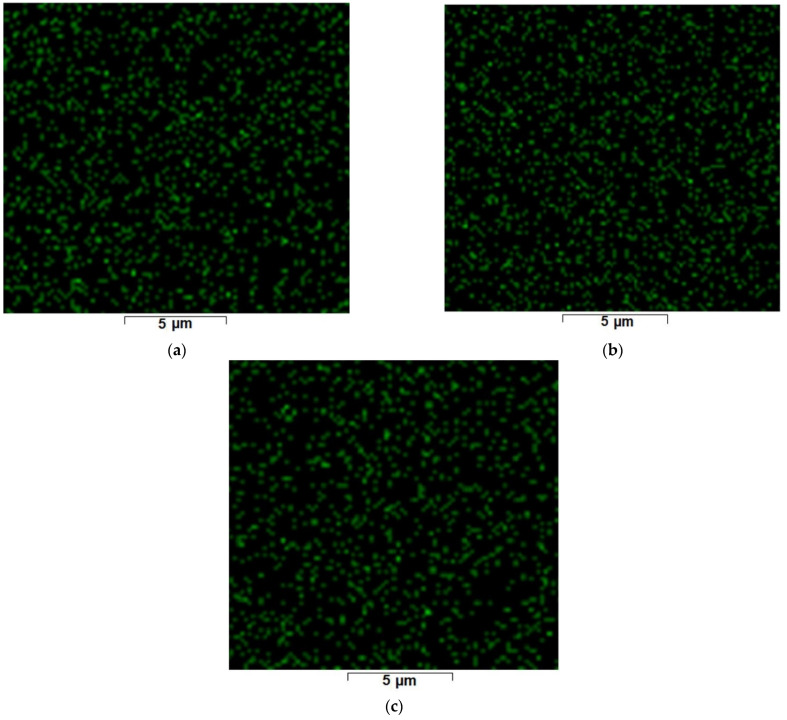
The energy dispersive spectroscopy analysis (EDAX) for the osmium nanoparticles-polypropylene hollow fibers composite membrane before (**a**); and after reduction (**b**); or oxidation (**c**) processes.

By scanning electron microscopy analysis (SEM) coupled with energy dispersive spectroscopy analysis (EDAX), it was possible to follow the distributions of osmium nanoparticles on the membranes recovered after the microfiltration of the respective reaction system: *tert-*butanol (Figure 9a); mass of reagents after the reduction process (Figure 9b); and mass of reagents after the oxidation process (Figure 9c).

The distribution of osmium on the surface of the target membrane was performed both before and after the redox processes (Figure 9a–c; see also Figure S6). The relatively uniform distribution on the membrane surface and the size of the nanoparticles were both remarkable. This uniform distribution of osmium nanoparticles on the processed membranes was due to the microfiltration process which, in addition to the disintegration effect, directed the nanoparticles on the surface and towards the interior of the pores, so that the map of nanoparticles reflected the pore distribution of the support membrane. The surface of 1 m^2^ represents a disadvantage for the study of nanoparticles, regardless of the analysis technique, due to dispersion (approx. 1.0–1.6 g/m^2^).

**Figure 10:**
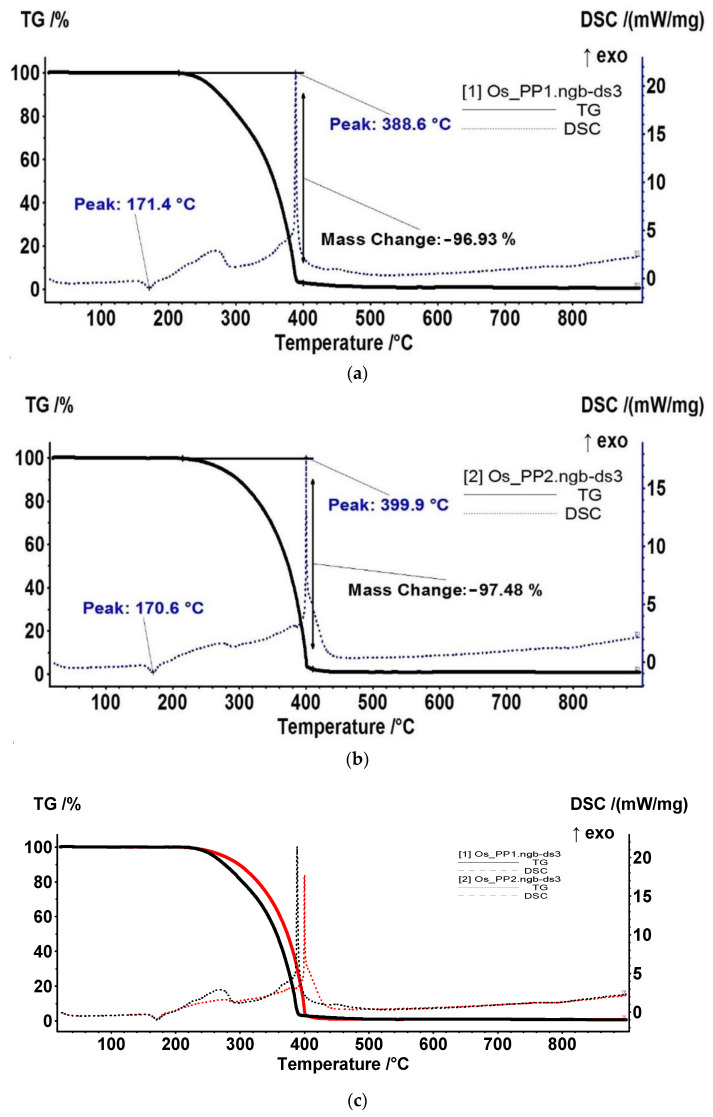
TG–DSC curves for osmium nanoparticles-polypropylene hollow fibers composite membranes: Os-PP1 (**a**); Os-PP2 (**b**); and their overlap (**c**).

The thermal analysis of the composite membranes gives information on the component’s interactions and membranes behavior at different temperatures. This is necessary in order to follow the performances in case of use at higher temperatures than the ambient one, and to fully characterize the composite material (Figure 10 and Table 2).

Both samples presented a similar degradation pathway but with some differences. The sample Os-PP2 had a higher thermal stability, the temperatures T_10_–T_50_ (at which 10-50% mass loss is recorded) being with ~20 °C higher.

The samples were stable up to 215 °C, with negligible or no mass loss recorded. On the DSC curve, the small endothermic peaks with onset at 159.7 °C and 160.4 °C, respectively, indicate the melting temperature. The higher value for the Os-PP2 sample indicates that the osmium compound was shielding the PP in a quantifiable measure.

After 215 °C, the complex degradative–oxidative process for the polymeric part took place. The samples were partially oxidized but also some decomposition took place, the evolved gases being composed of CO_2_ and H_2_O, but also saturated hydrocarbons and traces of CO and unsaturated hydrocarbons could be identified. Up to 300 °C, the decomposition and oxidation reactions were fairly equal, but after 300 °C, the oxidation reactions became predominant, with the burning taking place around 400 °C. The strong exothermic peak on DSC curve had maxima at 388.6 °C and 399.9 °C for Os-PP1 and Os-PP2, respectively.

**Figure 11:**
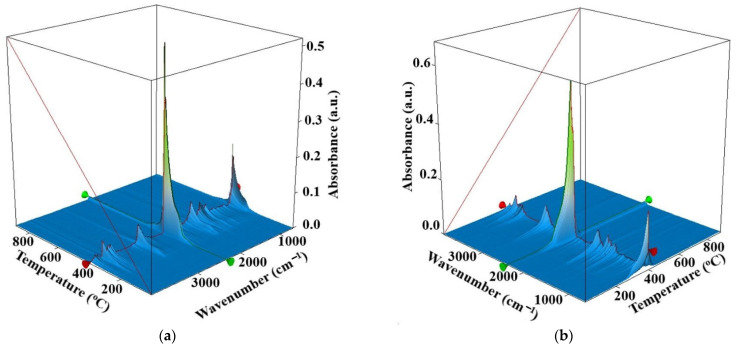
FTIR 3D chromatograms for evolved gases obtained for osmium nanoparticles-polypropylene hollow fiber composite membranes: Os-PP1 (**a**); and Os-PP2 (**b**).

In order to obtain more information on the thermal processes, the gases evolved from thermal analysis were investigated by FTIR during heating in the 20–900 °C range. Figure 11 present the 3D FTIR data for the evolved gases.

The 3D FTIR chromatograms for Os-PP1 (Figure 11a) and Os-PP2 (Figure 11b) allow the extraction of individual FTIR spectrum of the exhaust gases at temperatures of interest. In this case, the beginning of the thermal decomposition was taken into account, at 270 °C, in order to have related compositional information.

**Figure 12:**
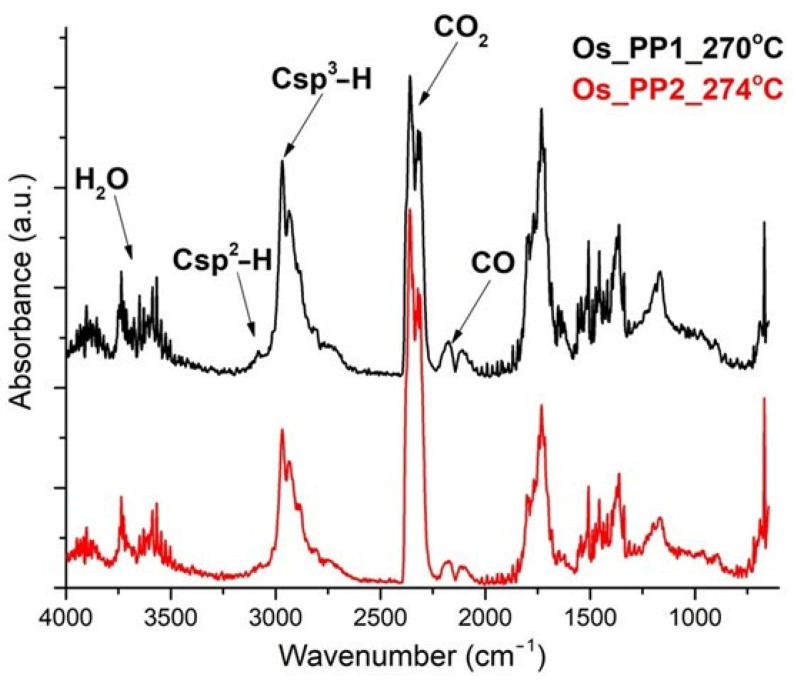
FTIR spectra of evolved gases at 270 °C for Os-PP1, and at 274 °C for Os-PP2.

In the FTIR spectra for Os-PP1 at 270 °C, and Os-PP2 at 274 °C (Figure 12), the presence of CO_2_ is mostly seen at 2355 cm^−1^, traces of CO at 2169 cm^−1^, water, but also the corresponding vibration of Csp^3^–H at 2964 cm^−1^. A small peak is observed at 3072 cm^−1^ (corresponding to Csp^2^–H fragments).

This indicates that the degradation of the composite membranes started as a complex process, in which both decomposition and oxidation of the polymeric matrix took place simultaneously.

**Figure 13:**
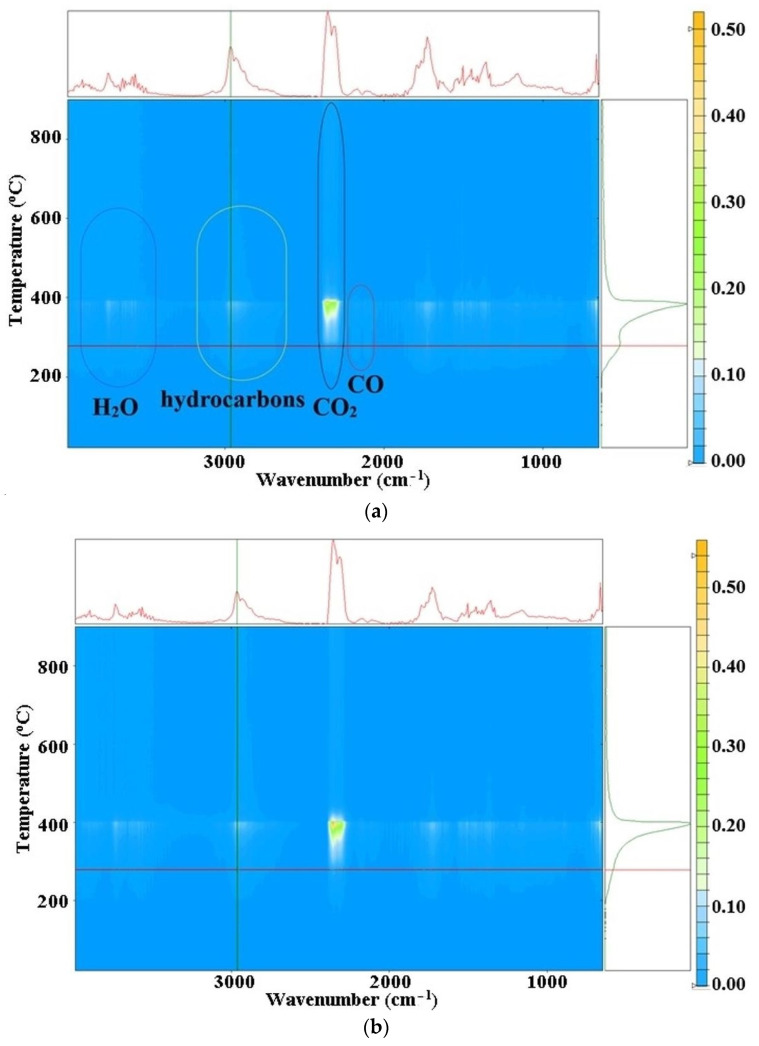
Bidimensional temperature/wavenumber projection for the 3D FTIR plot, indicating the temperature interval where various compounds were found in evolved gases from the thermal analysis of composite membranes: Os-PP1 (**a**); and Os-PP2 (**b**).

The bidimensional temperature/wavenumber projection of the 3D FTIR chromatogram for Os-PP1 (Figure 13a) can be used to visualize, at a glance, the temperature intervals where decomposition or oxidations took place. Figure 13a marks the areas where the vibration frequencies characteristic for water, CO_2_, CO, and hydrocarbons appeared, to highlight the elimination in time. The green, vertical line at 2964 cm^−1^ represents the release profile with temperature of the hydrocarbons. It was observed that the elimination profile of hydrocarbons by decomposition was different between Os-PP1 and Os-PP2. It can be stated that the differences between T_10_–T_50_ (Table 2) can be the subject of the study. Thus, in the case of Os-PP1, a more accentuated decomposition of polypropylene (PP) took place, while in the case of Os-PP2, this decomposition was lower in the 200–300 °C temperature interval.

### 3.2. Reduction of P-Nitrophenol with Molecular Hydrogen with Osmium Nanoparticles-Polypropylene Hollow Fibers Composite Membranes

The reduction of nitroderivatives is a reaction of ennobling of some intermediate compounds for multiple products of biological interest, and the nitrophenols are the most intensively studied target substances both for the accessibility of analytical monitoring and for their biomedical importance and impact on environment [24,47,48]. These two arguments are the basis for determination of the process performance of composite membranes based on osmium nanoparticles-polypropylene hollow fibers Os-PP1 and Os-PP2 in the reduction reaction of *p-*nitrophenol from lower aliphatic alcohol solutions with molecular hydrogen.

For the comparison of the results, a composite membrane of osmium nanoparticles-polypropylene hollow fibers Os-PP0 was tested, recently reported for reduction of 5-nitrobenzimidazole [64].

The reduction process was carried out according to the chemical reactions (4) and (5), but the equilibria (6) and (7) must also be considered, in which either the hydration water of osmium nanoparticles or the one resulting from reaction can participate.

(4) $$R–\mathrm{OH}\;+\;R–\mathrm{OH}\;⇄\;R–O^{-}+\;R–{\mathrm{OH}}_{2}^{+}$$

(5) $$\mathrm{HO}–C_{6}H_{4}–{\mathrm{NO}}_{2}+3H2_{\;}⇄\;\mathrm{HO}–C_{6}H_{4}–{\mathrm{NH}}_{2}+2H_{2}O$$

(6) $$\mathrm{HOH}+\mathrm{HOH}\;⇄\;\mathrm{HO}+H_{3}O^{+}$$

(7) $$H_{3}O^{+}+e^{–}\;⇄\;\;1/2H_{2}+H_{2}O$$

The reduction of nitro derivatives with osmium compounds is very well known [32,33,66], but the study of this process on osmium nanoparticles with hydrogen gas is much less approached, the in-situ generation of molecular hydrogen especially with sodium borohydride being preferred [67,68].

At the same time, the choice of the alcoholic reaction medium is sustained due to the low solubility of hydrogen gas in the aqueous medium [69].

**Figure 14:**
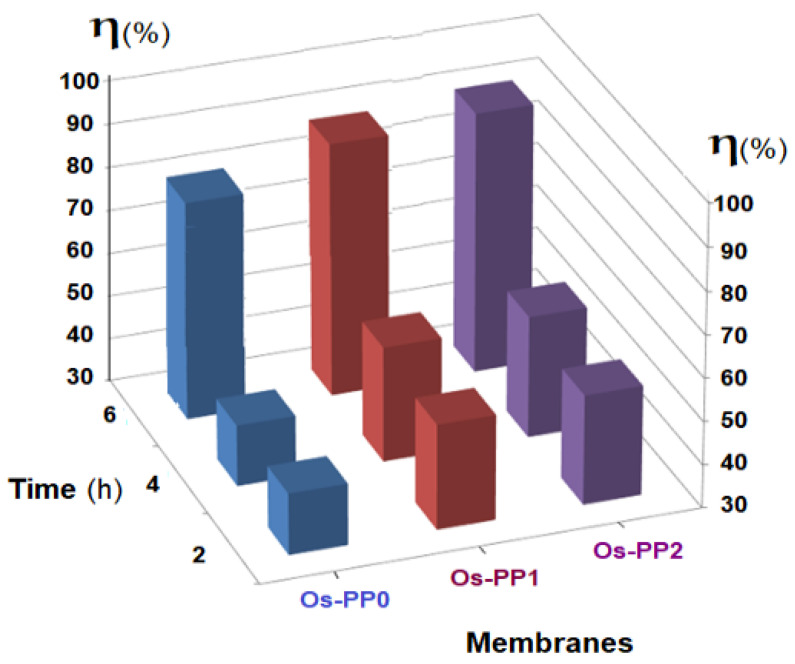
Influence of membrane type (Os-PP0, Os-PP1 and Os-PP2) on the conversion (*η* %), to the reduction of *p-*nitrophenol in *tert-*butyl alcohol solution as a function of operating time (h).

**Figure 15:**
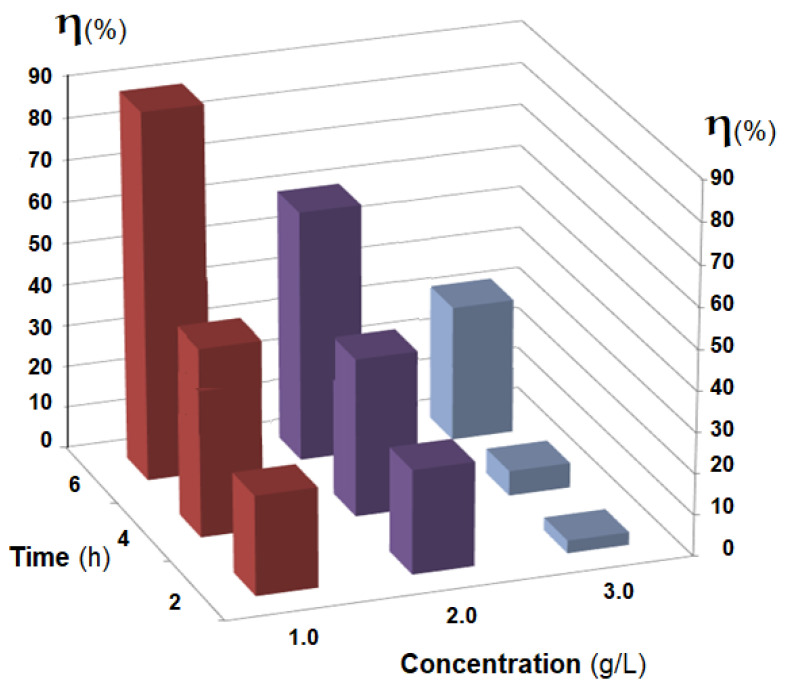
The influence, as a function of operating time (h), of *p-*nitrophenol concentration (g/L) on conversion (*η* %) to reduction of *p-*nitrophenol in *tert-*butyl alcohol solution.

**Figure 16:**
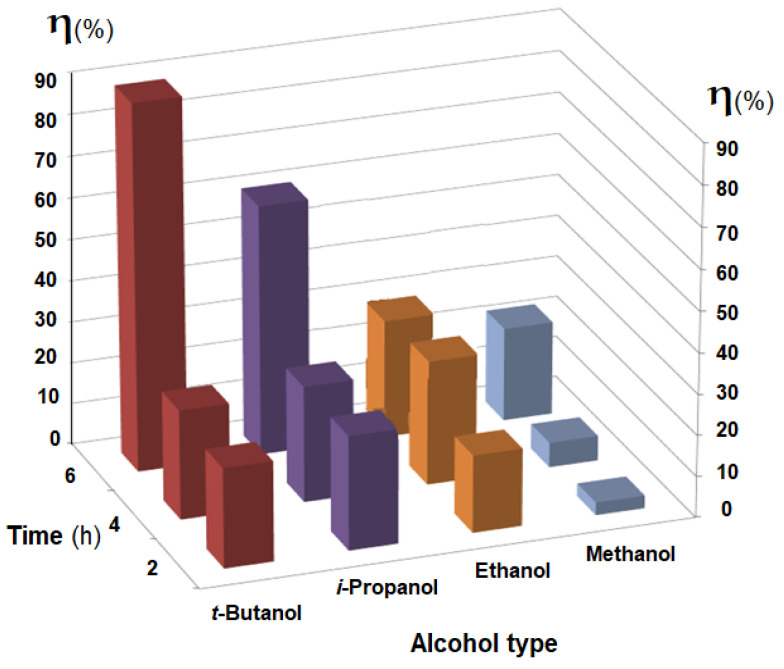
The influence of the nature of the primary alcohol (*t-*butanol, *i-*propanol, ethanol, or methanol) on the conversion (*η* %) to the reduction of *p-*nitrophenol in the solution, depending on the operating time (h).

The obtained results highlight the influence of the membrane type (Os-PP0, Os-PP1 and Os-PP2) (Figure 14), concentration of *p-*nitrophenol solution (Figure 15), and nature of the primary alcohol (Figure 16) over the conversion (*η* %), when reducing *p-*nitrophenol from alcoholic solution for the composite membranes of osmium nanoparticles-polypropylene hollow fibers (Os-PP).

*η* _Os-PP0_ < *η* _Os-PP1_ < *η* _Os-PP2_ (8)

In the membrane contactor (Figure 4), at a temperature of 25 °C, 10 L of 1g/L solution *p-*nitrophenol in *t-*butanol solution was introduced, which was recirculated at a flow rate of 0.100 L/min through the outside of the composite membranes of nanoparticles of osmium-polypropylene hollow fibers, while molecular hydrogen was continuously introduced into the system at a flow rate of 1 L/min through the membranes. The experiments performed for each type of composite membrane (Os-PP0, Os-PP1, and Os-PP2) for six hours showed that the evolution of hydrogenation was slow at the beginning of the operation interval, and the conversion value (*η* %) reached 75% (Os-PP0), 83% (Os-PP1), and 87% (Os-PP2) at six hours (Figure 14).

The conversion values are directly related to the osmium concentration at the membrane surface (Table 1) increasing in the following order:

Using the most performant composite membrane obtained (Os-PP2), the hydrogenation of *p-*nitrophenol was then followed, in concentrations of 1.0–3.0 g/L, under the same experimental conditions (Figure 15). The conversion of *p-*nitrophenol (*η* %) decreased with increasing concentration of *p-*nitrophenol in *t-*butanol solution. The results of the conversion (*η* %) at six hours of operation varies from 85% for the 1.0 g/L solution, 56% for the 2.0 g/L solution, and 28% for the 3.0 g/L solution. For a high conversion, it will be necessary to either operate with dilute solutions or increase the working time.

The type of alcohol, used as solvent, significantly influenced the conversion of *p-*nitrophenol for the tested membrane (Os-PP2) (Figure 16). The results obtained for a *p-*nitrophenol solution of 1.0 g/L, under the previous experimental conditions, at six hours of operation, showed a conversion (*η* %) of 85% for *t-*butanol, 57% for *i-*propanol, 24% for ethanol, and 19% for methanol.

The study performed on the reduction reaction of *p-*nitrophenol from solutions of lower aliphatic alcohols, with molecular hydrogen, on composite membranes of osmium nanoparticles-polypropylene hollow fibers (Os-PP) allowed the establishment of the following parameters:

- Composite membrane of osmium nanoparticles-polypropylene hollow fibers: Os-PP2 (7.63% atomic bone on the surface),
- Operating time: six hours,
- Concentration of *p-*nitrophenol in *t-*butanol: 1 g/L,
- Primary alcohol: *t-*butanol.

### 3.3. Oxidation of 10-Undecylenic Acid on Osmium Nanoparticles-Polypropylene Hollow Fiber Composite Membranes Using Molecular Oxygen

The 10-undecylenic acid is an accessible substance with multiple industrial applications, in sports biomedicine but also as membrane material (spacer). The oxidation of this compound is of particular interest for technical applications, but especially biomedical, because its properties can be altered when the athletes who use it bring it into contact with various powders (talc, zinc oxide, titanium oxide) used for adhesion and solar UV rays [70,71,72,73].

**Figure 17:**
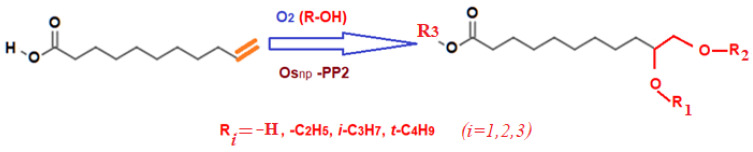
Scheme of molecular oxygen oxidation of 10-undecylenic acid on osmium nanoparticles-polypropylene hollow fibers (Os-PP2) composite membrane.

In the present case, the oxidation of 10-undecylenic acid was achieved from alcoholic solution on osmium nanoparticles-polypropylene hollow fibers composite membranes, using molecular oxygen (Figure 17). The reaction of oxidation performed in the installation depicted in Figure 4 used an alcoholic solution (ethanol, *i-*propanol, and *t-*butanol) of 10-undecylenic acid of concentration of 1.0, 3.0, and 5.0 g/L at a volume of 10 L and a recirculation flow of 0.100 L/min, through the contactor membrane system, through the outside of the osmium-polypropylene hollow fiber composite membranes (Os-PP2). Molecular oxygen was introduced through membranes, at a flow rate of 1 L/minute, a temperature of 25 °C, and a pressure of 1 atm. The operating time was set at a maximum of five hours.

**Figure 18:**
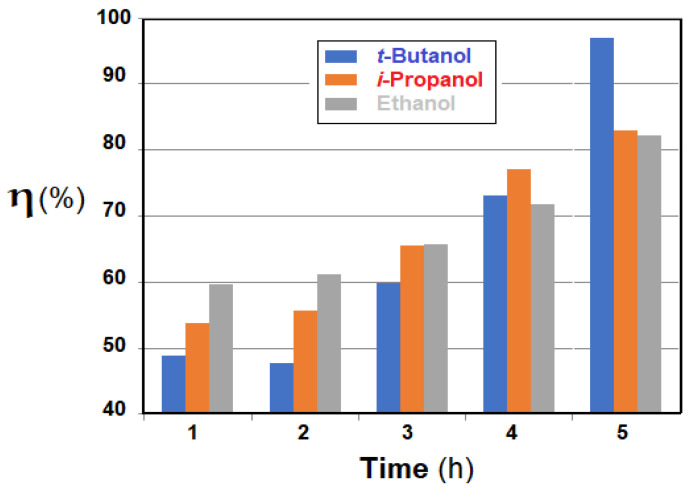
Conversion (*η* %), as a function of time, of 10-undecylenic acid after molecular oxygen oxidation on the osmium nanoparticles-polypropylene hollow fibers (Os-PP2) composite membrane, for selected primary alcohols.

Figure 18 shows the results of 10-undecylenic acid oxidation, depending on time and nature of the primary alcohol used. The conversion (*η* %) of 10-undecylenic grew from 48% to 97% during five hours for *t-*butanol, and from 60% to 82% for ethanol. The conversion for *i-*propanol fell between the other two solvents. The conversion began more abruptly for the solvent with lower molecular weight, but towards the end of the operation, the order of conversion in the presence of the three alcohols was reversed. Most likely, this aspect of the conversion was determined by the two stages of the process: double-bond oxidation followed by alcoholysis.

**Figure 19:**
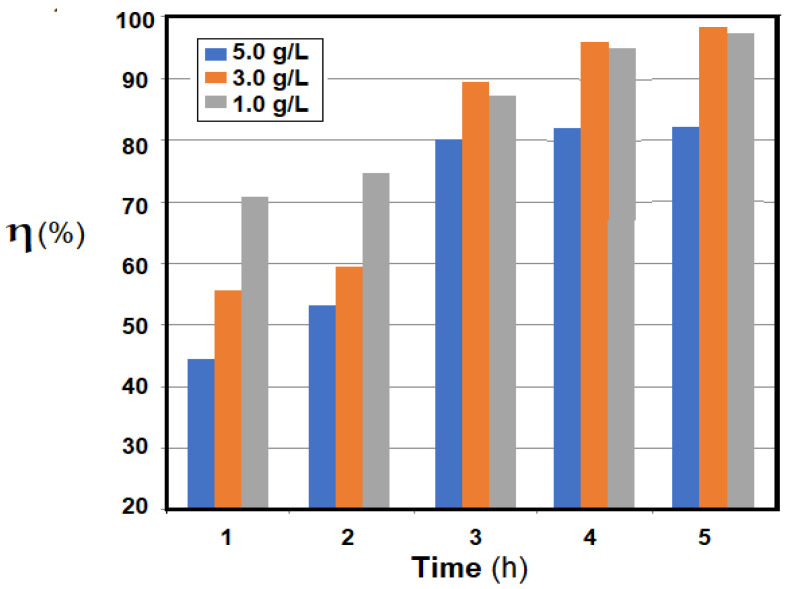
Conversion (*η* %), as a function of time, of 10-undecylenic acid of variable concentration, after molecular oxygen oxidation on the osmium nanoparticles-polypropylene hollow fibers (Os-PP2) composite membrane.

To study the influence of 10-undecylenic acid concentration in the alcohol solution as a function of time, the solvent that showed the maximum conversion at five hours of operation, *t-*butanol, was chosen (Figure 19).

The conversion (*η* %) as a function of time for 10-undecylenic acid of variable concentration (1.0, 3.0 and 5.0 g/L) at oxidation with molecular oxygen on the osmium nanoparticles-polypropylene hollow fibers (Os-PP2) composite membrane showed that at high concentrations, the conversion was lower at the beginning of oxidation, but during the process, the conversions approached each other, reaching values of over 80% for the entire chosen concentration range after five hours.

The oxidation of 10-undecylenic acid follows a probable three-stage mechanism:

1. Through the intervention of osmium nanoparticles, oxygen is physically-chemically linked to the double-bond;
2. The alcohol (mostly in the environment) produces the alcoholysis of the osmic acid ester producing a variety of reaction products (the reaction was modified in manuscript according to these data); and
3. The products are released into the environment.

The complex qualitative composition of the reaction mass, determined by chromatographic separation and FTIR spectroscopy identification of the components, showed that the glycolic compound obtained was majority (R_1_, R_2_, and R_3_ being the hydrogen atoms), but also alkylated compounds with ethyl, propyl, and butyl (in various positions) were detected (see Table S1).

A very special aspect of our research is the problem of loss of osmium and/or osmium nanoparticles in the reaction medium. Beyond the high economic value of osmium, the toxicity of its compounds (especially osmium tetroxide) requires both operational safety measures and rigorous control of operations. For the research carried out in this study, osmium nanoparticles were permanently operated in reaction space outside the fibers. To avoid the loss of osmium nanoparticles from the reaction compartment, the alcoholic reaction mass was extracted from the membrane module by vacuum suction inside the polypropylene hollow fiber membranes. At the same time, the reaction compartment was subjected to a nitrogen gas flow after each reduction operation (hydrogenation) before the recovery of the reaction mass (See Figure S7). After the oxidation process, the reaction compartment underwent three stages of tartar: with nitrogen gas to remove residual oxygen, with hydrogen (at least two hours to reduce any traces of osmium tetroxides), and then with nitrogen to remove residual hydrogen. The filtration characteristics of polypropylene hollow fiber membranes [56,57,58,59,60,61,62,63,64] ensured the complete retention of osmium nanoparticles in the reaction compartment which could be followed by established analytical techniques [74,75,76,77,78].

## 4. Conclusions

This paper presented the preparation and characterization of a composite membrane of active metal nanoparticle–polymer support type, based on osmium nanoparticles obtained in situ on a polypropylene hollow fiber membrane. The osmium nanoparticles were generated from a solution of osmium tetroxide of different concentrations in *tert-*butyl alcohol, by reduction with molecular hydrogen, in a contactor with polypropylene membrane.

The osmium nanoparticles-polypropylene hollow fibers (Os-PP) composite membranes were characterized morphologically and structurally by scanning electron microscopy (SEM), thermal analysis, surface analysis, and Fourier transform infrared (FTIR) spectroscopy.

The performance of osmium nanoparticles-polypropylene hollow fibers (Os-PP2) composite membranes was tested during the processes of reduction or oxidation of two compounds of technological and biomedical interests (*p-*nitrophenol and 10-undecylenic acid) from lower saturated alcohol solutions.

The results obtained showed that the reduction of *p-*nitrophenol with molecular hydrogen on composite membranes based on osmium nanoparticles-polypropylene hollow fibers (Os-PP) can be performed with conversions of about 90%, under the following conditions:

- Composite membrane of osmium nanoparticles-polypropylene hollow fibers: Os-PP2 (7.63% atomic Os on the surface),
- Operating time: six hours,
- The concentration *p-*nitrophenol in *t-*butanol: 1 g/L,
- Primary alcohol: *t-*butanol.

Oxidation of 10-undecylenic acid with molecular oxygen on composite membranes of osmium nanoparticles-polypropylene hollow fibers (Os-PP) took place with conversions of over 80% of solutions in *t-*butanol of concentration 1–5 g/L, at an operating time of five hours. The composition of the reaction mass to the oxidation of 10-undecylenic acid is complex and requires further studies.

To avoid the loss of osmium nanoparticles from the reaction compartment outside the fibers of the membrane module, the alcoholic reaction mass was extracted from the membrane module by vacuum suction inside the polypropylene hollow fiber membranes.

## Figures and Tables

**Table 1:** Energy dispersive spectroscopy analysis (EDAX) for the osmium-polymer membranes.

Composite Membranes	(Os-PP0)			(Os-PP1)			(Os-PP2)		
Surface Composition	Weight (%)	Atomic (%)	Error (%)	Weight (%)	Atomic (%)	Error (%)	Weight (%)	Atomic (%)	Error (%)
C K	49.36	90.32	9.44	43.22	88.09	9.81	39.90	85.94	9.98
O K	2.89	4.05	29.4	3.29	5.03	31.05	3.98	6.43	33.41
Os L	47.75	5.63	11.12	53.49	6.88	13.32	56.12	7.63	14.03

**Table 2:** Thermal characteristics of the obtained composite membranes.

Sample	T_10_	T_20_	T_30_	T_40_	T_50_	Melting Onset	Exo Burning
	(°C)	(°C)	(°C)	(°C)	(°C)	(°C)	(°C)
Os-PP1	276.1	303.8	327.2	344.2	356.8	159.7	388.6
Os-PP2	298.3	327.6	347.7	362.3	373.5	160.4	399.9

## Supplementary Materials

The following are available online at https://www.mdpi.com/article/10.3390/nano11102526/s1, Figure S1: The appearances of crystallized metallic osmium and osmium powder, Figure S2: Images with the solution of osmium tetroxide in tert-butanol, the obtained metallic osmium and the osmium powder retained on a microporous membrane, Figure S3: Images with the bottle in which the osmium tetroxide was kept tightly sealed and the osmium powder obtained, Figure S4: Scanning electron microscopy (SEM) images of polypropylene hollow fiber membrane support, Figure S5: X-ray diffraction diagrams for polypropylene hollow fiber membrane dust, Figure S6: Scanning electron microscopy (SEM) and energy dispersive spectroscopy analysis (EDAX) on polypropylene hollow fiber membrane support, Figure S7: The specific safety operations on reactional processes and the obtained membranes, Table S1: The composition of the reaction mass to the oxidation with molecular oxygen of 10-undecylenic acid on osmium-polypropylene hollow fiber composite membranes.

## References

[1] Liao Z., Zhu J., Li X., Van der Bruggen B. (2021). Regulating composition and structure of nanofillers in thin film nanocomposite (TFN) membranes for enhanced separation performance: A critical review. Sep. Purif. Technol..

[2] Zhu J., Hou J., Uliana A., Zhang Y., Tian M., Van der Bruggen B. (2018). The rapid emergence of two-dimensional nanomaterials for high-performance separation membranes. J. Mater. Chem. A.

[3] Abdelsamad A.M.A., Khalil A.S.G., Ulbricht M. (2018). Influence of controlled functionalization of mesoporous silica nanoparticles as tailored fillers for thin-film nanocomposite membranes on desalination performance. J. Membr. Sci..

[4] Ang M.B.M.Y., Pereira J.M., Trilles C.A., Aquino R.R., Huang S.-H., Lee K.-R., Lai J.-Y. (2019). Performance and antifouling behavior of thin-film nanocomposite nanofiltration membranes with embedded silica spheres. Sep. Purif. Technol..

[5] Lai G.S., Lau W.J., Goh P.S., Ismail A.F., Tan Y.H., Chong C.Y., Krause-Rehberg R., Awad S. (2018). Tailor-made thin film nanocomposite membrane incorporated with graphene oxide using novel interfacial polymerization technique for enhanced water separation. Chem. Eng. J..

[6] Firouzjaei M.D., Shamsabadi A.A., Aktij S.A., Seyedfour S.F., Sharifian Gh M., Rahimpour A., Esfahani M.R., Ulbricht M., Soroush M. (2018). Exploiting synergetic effects of graphene oxide and a silver-based metal-organic M.R.; framework to enhance antifouling and anti-biofouling properties of thin-film nanocomposite membranes. ACS Appl. Mater. Interfaces.

[7] Zhang A., Zhang Y., Pan G., Xu J., Yan H., Liu Y. (2017). In situ formation of copper nanoparticles in carboxylated chitosan layer: Preparation and characterization of surface modified TFC membrane with protein fouling resistance and long-lasting antibacterial properties. Sep. Purif. Technol..

[8] Upadhyaya L., Semsarilar M., Quemener D., Fernández-Pacheco R., Martinez G., Coelhoso I.M., Nunes S.P., Crespo J.G., Mallada R., Portugal C.A.M. (2021). Block Copolymer-Based Magnetic Mixed Matrix Membranes—Effect of Magnetic Field on Protein Permeation and Membrane Fouling. Membranes.

[9] Kuroda K., Ishida T., Haruta M. (2009). Reduction of 4-nitrophenol to 4-aminophenolover Au nanoparticles deposited on PMMA. J. Mol. Catal. A Chem..

[10] Koga H., Kitaoka T. (2011). One-step synthesis of gold nanocatalysts on a micro-structured paper matrix for the reduction of 4-nitrophenol. Chem. Eng. J..

[11] Dong Z., Le X., Dong C., Zhang W., Li X., Ma J. (2015). Ni@Pd core-shell nanoparticles modified fibrous silica nanospheres as highly efficient and recoverable catalyst for reduction of 4-nitrophenol and hydrodechlorination of 4-chlorophenol. Appl. Catal. B Environ..

[12] Woo S.T., Yun T., Kwak S.-Y. (2018). Fouling-resistant microfiltration membrane modified with magnetite nanoparticles by reversible conjunction. Sep. Pur. Technol..

[13] Ghaemi N., Madaeni S.S., Daraei P., Rajabi H., Zinadini S., Alizadeh A., Heydari R., Beygzadeh M., Ghouzivand S. (2015). Polyethersulfone membrane enhanced with iron oxide nanoparticles for copper removal from water: Application of new functionalized Fe3O4 nanoparticles. Chem. Eng. J..

[14] Agbaje T.A., Al-Gharabli S., Mavukkandy M.O., Kujawa J., Arafat H.A. (2018). PVDF/magnetite blend membranes for enhanced flux and salt rejection in membrane distillation. Desalination.

[15] Harish S., Mathiyarasu J., Phani K.L.N., Yegnaraman V. (2009). Synthesis of conducting polymer supported Pd nanoparticles in aqueous medium and catalytic activity towards 4-nitrophenol reduction. Catal. Lett..

[16] Khalil A.M., Georgiadou V., Guerrouache M., Mahouche-Chergui S., Dendrinou-Samara C., Chehimi M.M., Carbonnier B. (2015). Gold- decorated polymeric monoliths: In-situ vs. ex-situ immobilization strategies and flow through catalytic applications towards nitrophenols reduction. Polymer.

[17] Macanás J., Ouyang L., Bruening M.L., Muñoz M., Remigy J.-C., Lahitte J.-F. (2010). Development of polymeric hollow fiber membranes containing catalytic metal nanoparticles. Catal. Today.

[18] Wang C., Yin J., Han S., Jiao T., Bai Z., Zhou J., Zhang L., Peng Q. (2019). Preparation of Palladium Nanoparticles Decorated Polyethyleneimine/Polycaprolactone Composite Fibers Constructed by Electrospinning with Highly Efficient and Recyclable Catalytic Performances. Catalysts.

[19] Rajakumaran R., Kumar M., Chetty R. (2020). Morphological effect of ZnO nanostructures on desalination performance and antibacterial activity of thin-film nanocomposite (TFN) membrane. Desalination.

[20] Wang J., Wang Y., Zhu J., Zhang Y., Liu J., van der Bruggen B. (2017). Construction of TiO_2_@graphene oxide incorporated antifouling nanofiltration membrane with elevated filtration performance. J. Membr. Sci..

[21] Liu C., Faria A.F., Ma J., Elimelech M. (2017). Mitigation of biofilm development on thin film composite membranes functionalized with zwitterionic polymers and silver nanoparticles. Environ. Sci. Technol..

[22] Mehrabi Z., Taheri-Kafrani A., Asadnia M., Razmjou A. (2020). Bienzymatic modification of polymeric membranes to mitigate biofouling. Sep. Purif. Technol..

[23] Hradil J., Krystl V., Hrabanek P., Bernauer B., Kocirık M. (2004). Heterogeneous membranes based on polymeric adsorbents for separation of small molecules. React. Funct. Polym..

[24] Yin J., Zhan F., Jiao T., Deng H., Zou G., Bai Z., Zhang Q., Peng Q. (2020). Highly efficient catalytic performances of nitro compounds via hierarchical PdNPs-loaded MXene/polymer nanocomposites synthesized through electrospinning strategy for wastewater treatment. Chin. Chem. Lett..

[25] Wang H., Dong Z., Na C. (2013). Hierarchical carbon nanotube membrane-supported gold nanoparticles for rapid catalytic reduction of p-nitrophenol. ACS Sustain. Chem. Eng..

[26] Wu W., Liu G., Liang S., Chen Y., Shen L., Zheng H., Yuan R., Hou Y., Wu L. (2012). Efficient visible-light-induced photocatalytic reduction of 4-nitroaniline to p-phenylenediamine over nanocrystalline PbBi2Nb2O9. J. Catal..

[27] Le X., Dong Z., Li X., Zhang W., Le M., Ma J. (2015). Fibrous nano-silica supported palladium nanoparticles: An efficient catalyst for the reduction of 4-nitrophenol and hydrodechlorination of 4-chlorophenol under mild conditions. Catal. Commun..

[28] Fang Y., Wang E. (2013). Simple and direct synthesis of oxygenous carbon supported palladium nanoparticles with high catalytic activity. Nanoscale.

[29] Bazhenov S.D., Bildyukevich A.V., Volkov A.V. (2018). Gas-liquid hollow fiber membrane contactors for different applications. Fibers.

[30] Sirkar K.K., Shanbhag P.V., Kovvali A.S. (1999). Membrane in a Reactor:  A Functional Perspective. Ind. Eng. Chem. Res..

[31] de Pedro Z.M., Diaz E., Mohedano A.F., Casas J.A., Rodriguez J. (2011). Compared activity and stability of Pd/Al2O3 and Pd/AC catalysts in 4-chlorophenol hydrodechlorination in different pH media. J. Appl. Catal. B Environ..

[32] Borja-Arco E., Castellanos R.H., Uribe-Godínez J., Altamirano-Gutiérrez A., Jiménez-Sandoval O. (2009). Osmium-ruthenium carbonyl clusters as methanol tolerant electrocatalysts for oxygen reduction. J. Power Sources.

[33] Bolitho E.M., Coverdale J.P.C., Bridgewater H.E., Clarkson G.J., Quinn P.D., Sanchez-Cano C., Sadler P.J. (2021). Tracking Reactions of Asymmetric Organo-Osmium Transfer Hydrogenation Catalysts in Cancer Cells. Angew. Chem. Int. Ed..

[34] Adams C.W.M., Abdulla Y.H., Bayliss O.B. (1967). Osmium tetroxide as a histochemical and histological reagent. Histochemie.

[35] Heller A. (2006). Electron-conducting redox hydrogels: Design, characteristics and synthesis. Curr. Opin. Chem. Biol..

[36] Uribe-Godínez J., Castellanos E., Borja-Arco R.H., Altamirano-Gutiérrez A., Jiménez-Sandoval O. (2008). Novel osmium-based electrocatalysts for oxygen reduction and hydrogen oxidation in acid conditions. J. Power Sources.

[37] Zhang H., Liu G., Shi L., Ye J. (2018). Single-Atom Catalysts: Emerging Multifunctional Materials in Heterogeneous Catalysis. Adv. Mat..

[38] Sharpless K.B., Amberg W., Bennani Y.L., Crispino G.A., Hartung J., Jeong K.S., Kwong H.L., Morikawa K., Wang Z.M. (1992). The osmium-catalyzed asymmetric dihydroxylation: A new ligand class and a process improvement. J. Org. Chem..

[39] Kolb H.C., Van Nieuwenhze M.S., Sharpless K.B. (1994). Catalytic Asymmetric Dihydroxylation. Chem. Rev..

[40] Ma L., Abney C., Lin W. (2009). Enantioselective catalysis with homochiral metal–organic frameworks. Chem. Soc. Rev..

[41] Yoon T.P., Jacobsen E.N. (2003). Privileged Chiral Catalysts. Science.

[42] Keith L., Telliard W. (1979). Priority Pollutants. Environ. Sci. Technol..

[43] Nasrollahzadeh M., Jaleh B., Fakhri P., Zahraei A., Ghadery E. (2015). Synthesis and catalytic activity of carbon supported copper nanoparticles for the synthesis of aryl nitriles and 1,2,3-triazoles. RSC Adv..

[44] Kristanti R.A., Toyama T., Hadibarata T., Tanaka Y., Mori K. (2014). Sustainable removal of nitrophenols by rhizoremediation using four strains of bacteria and giant duckweed (*Spirodela polyrhiza*). Water. Air. Soil Pollut..

[45] Xiong Z., Zhang H., Zhang W., Lai B., Yao G. (2019). Removal of Nitrophenols and their Derivatives by Chemical Redox: A Review. Chem. Eng. J..

[46] Jaleh B., Karami S., Sajjadi M., Feizi B., Azizian S., Nasrollahzadeh M., Varma R.S. (2020). Laser-assisted preparation of Pd nanoparticles on carbon cloth for the degradation of environmental pollutants in aqueous medium. Chemosphere.

[47] Vassilev D., Petkova N., Tumbarski Y., Koleva M., Denev P. (2020). Application of the principles of “green chemistry” for the synthesis of 10-undecylenic aliphatic esters with antimicrobial activity. J. Renew. Mater..

[48] Yasa S., Cheguru S., Krishnasamy S., Korlipara P., Rajak A., Penumarthy V. (2017). Synthesis of 10-undecenoic acid based C22-dimer acid esters and their evaluation as potential lubricant basestocks. Ind. Crops Prod..

[49] Raku T., Kitagawa M., Shimakawa H., Tokiwa Y. (2003). Enzymatic synthesis of hydrophilic undecylenic acid sugar esters and their biodegradability. Biotechnol. Lett..

[50] Cavalcante I.M., Rocha N.R.D.C., de Brito D.H.A., Schuller A.P.D., Camara Neto J.F., de Morais S.M., de Luna F.M.T., Schanz M.T.G.F., Maier M.E., Ricardo N.M.P.S. (2019). Synthesis and characterization of novel polyol esters of undecylenic acid as ecofriendly lubricants. J. Am. Oil Chem Soc..

[51] Huerta-Ángeles G., Brandejsová M., Kopecká K., Ondreáš F., Medek T., Židek O., Kulhánek J., Vagnerová H., Velebný V. (2020). Synthesis and Physicochemical Characterization of Undecylenic Acid Grafted to Hyaluronan for Encapsulation of Antioxidants and Chemical Crosslinking. Polymers.

[52] Ghimpusan M., Nechifor G., Din I.S., Nechifor A.C., Passeri P. (2016). Application of Hollow Fibre Membrane Bioreactor Instead of Granular Activated Carbon Filtration for Treatment of Wastewater from Car Dismantler Activity. Mat. Plast..

[53] Din I.S., Cimbru A.M., Rikabi A.A.K.K., Tanczos S.K., Ticu (Cotorcea) S., Nechifor G. (2018). Iono-molecular Separation with Composite Membranes VI. Nitro-phenol separation through sulfonated polyether ether ketone on capillary polypropylene membranes. Rev. Chim. Buchar..

[54] Ghimpusan M., Nechifor G., Nechifor A.C., Dima S.O., Passeri P. (2017). Case studies on the physical-chemical parameters’ variation during three different purification approaches destined to treat wastewaters from food industry. J. Environ. Manag..

[55] Dimulescu I.A., Nechifor A.C., Bărdacă C., Oprea O., Paşcu D., Totu E.E., Albu P.C., Nechifor G., Bungău S.G. (2021). Accessible Silver-Iron Oxide Nanoparticles as a Nanomaterial for Supported Liquid Membranes. Nanomaterials.

[56] Nechifor A.C., Cotorcea S., Bungău C., Albu P.C., Pașcu D., Oprea O., Grosu A.R., Pîrțac A., Nechifor G. (2021). Removing of the Sulfur Compounds by Impregnated Polypropylene Fibers with Silver Nanoparticles-Cellulose Derivatives for Air Odor Correction. Membranes.

[57] Grosu A.R., Nafliu I.M., Din I.S., Cimbru A.M., Nechifor G. (2020). Neutralization with simultaneous separation of aluminum and copper ions from condensed water through capillary polypropylene and cellulose. UPB Sci. Bull. Ser. B Chem. Mater. Sci..

[58] Nechifor A.C., Goran A., Grosu V.-A., Bungău C., Albu P.C., Grosu A.R., Oprea O., Păncescu F.M., Nechifor G. (2021). Improving the Performance of Composite Hollow Fiber Membranes with Magnetic Field Generated Convection Application on pH Correction. Membranes.

[59] Nechifor A.C., Pîrțac A., Albu P.C., Grosu A.R., Dumitru F., Dimulescu I.A., Oprea O., Pașcu D., Nechifor G., Bungău S.G. (2021). Recuperative Amino Acids Separation through Cellulose Derivative Membranes with Microporous Polypropylene Fiber Matrix. Membranes.

[60] Diaconu I., Nechifor G., Nechifor A.C., Ruse E., Totu E.E. (2009). Membranary techniques used at the separation of some phenolic compounds from aqueous media, UPB Scientific Bulletin. Ser. B Chem. Mater. Sci..

[61] Szczepański P., Diaconu I. (2012). Transport of p-nitrophenol through an agitated bulk liquid membrane. Sep. Sci. Technol..

[62] Koter S., Szczepański P., Mateescu M., Nechifor G., Badalau L., Koter I. (2013). Modeling of the cadmium transport through a bulk liquid membrane. Sep. Purif. Technol..

[63] Diaconu I., Gîrdea R., Cristea C., Nechifor G., Ruse E., Totu E.E. (2010). Removal and recovery of some phenolic pollutants using liquid membranes. Rom. Biotechnol. Lett..

[64] Nechifor A.C., Goran A., Grosu V.-A., Pîrțac A., Albu P.C., Oprea O., Grosu A.R., Pașcu D., Păncescu F.M., Nechifor G. (2021). Reactional Processes on Osmium-Polymeric Membranes for 5-Nitrobenzimidazole Reduction. Membranes.

[65] Bărdacă Urducea C., Nechifor A.C., Dimulescu I.A., Oprea O., Nechifor G., Totu E.E., Isildak I., Albu P.C., Bungău S.G. (2020). Control of Nanostructured Polysulfone Membrane Preparation by Phase Inversion Method. Nanomaterials.

[66] George A., Selvan D., Mandal S. (2017). Catalytic Reduction of Toxic Nitroarenes in Aqueous Medium Using Worm-Like Rhodium Nanoparticles. Chem. Sel..

[67] Huang T., Fu Y., Peng Q., Yu C., Zhu J., Yu A., Wang X. (2019). Catalytic hydrogenation of p-nitrophenol using a metal-free catalyst of porous crimped graphitic carbon nitride. Appl. Surf. Sci..

[68] Lu H., Yin H., Liu Y., Jiang T., Yu L. (2008). Influence of support on catalytic activity of Ni catalysts in p-nitrophenol hydrogenation to p-aminophenol. Catal. Commun..

[69] Herskowitz M., Wisniak J., Skladman L. (1983). Hydrogen solubility in organic liquids. J. Chem. Eng. Data.

[70] Mionić Ebersold M., Petrović M., Fong W.-K., Bonvin D., Hofmann H., Milošević I. (2018). Hexosomes with Undecylenic Acid Efficient against Candida Albicans. Nanomaterials.

[71] Liakos I.L., Holban A.M., Carzino R., Lauciello S., Grumezescu A.M. (2017). Electrospun Fiber Pads of Cellulose Acetate and Essential Oils with Antimicrobial Activity. Nanomaterials.

[72] Shi D., Zhao Y., Yan H., Fu H., Shen Y., Lu G., Mei H., Qiu Y., Li D., Liu W. (2016). Antifungal effects of undecylenic acid on the biofilm formation of Candida albicans. Int. J. Clin. Pharmacol. Ther..

[73] Petrović M., Bonvin D., Hofmann H., Mionić Ebersold M. (2018). Fungicidal PMMA-Undecylenic Acid Composites. Int. J. Mol. Sci..

[74] Zanje S.B., Kokare A.N., Suryavanshi V.J., Waghmode D.P., Joshi S.S., Anuse M.A. (2016). Development of a reliable analytical method for the precise extractive spectrophotometric determination of osmium(VIII) with 2-nitrobenzaldehydethiocarbohydrazone: Analysis of alloys and real sample. Spectrochim. Acta Part A Mol. Biomol. Spectrosc..

[75] Tabatabaee M., Bagheri H., Shahvazian M. (2010). Application of thionine dye for highly sensitive and selective catalytic kinetic determination of osmium. Prog. Color Colorants Coat..

[76] Tang B., Zhang H., Wang Y. (2005). Flow injection kinetic spectrofluorimetric determination of trace amounts of osmium. Spectrochim. Acta Part A.

[77] Suzuki T., Miyada M., Ohta K., Kaneco S., Mizuno T. (1998). Determination of osmium in waste water by graphite furnace atomic absorption spectrometry. Mikrochim. Acta.

[78] Gregoire D.C. (1990). Sample introduction techniques for the determination of osmium isotope ratios by inductively coupled plasma mass spectrometry. Anal. Chem..

