# Supplementary material for: Osmium Nanoparticles-Polypropylene Hollow Fiber Membranes Applied in Redox Processes
Source: Nanomaterials (Basel). 2021 Sep 27;11(10):2526. doi: 10.3390/nano11102526 (PMC8537536; doi:10.3390/nano11102526)
Supplement: Supplementary file 1 [file nanomaterials-11-02526-s001.zip › nanomaterials-1327969-supplementary.pdf]

nanomaterials

Supplementary Material

Osmium Nanoparticles–Polypropylene Hollow Fiber Membranes Applied in Redox Processes

Gheorghe Nechifor [1], Florentina Mihaela Păncescu [1], Alexandra Raluca Grosu [1], Paul Constantin Albu [2,*], Ovidiu Oprea [3], Szidonia-Katalin Tanczos [4], Constantin Bungău [5], Vlad-Alexandru Grosu [6,*], Andreia Pîrțac [1], Aurelia Cristina Nechifor [1]

1. *Osmium removal from osmium tetroxide*

The metallic osmium (Os) is one of the least reactive elements (Figure S1a), however the European Commission classifies it as a risk class, as toxic and dangerous substance (Classification according to Regulation EC No 1272/2008). This classification is determined by the high affinity of osmium for oxygen, especially when it is in powder form (Figure S1b), when the osmium tetroxide (OsO_4) is produced. OsO_4 is extremely reactive, interacting with organic and biological chemicals, which makes it irritating both to sensitive parts of humans and to inhalation or ingestion (equation S1).

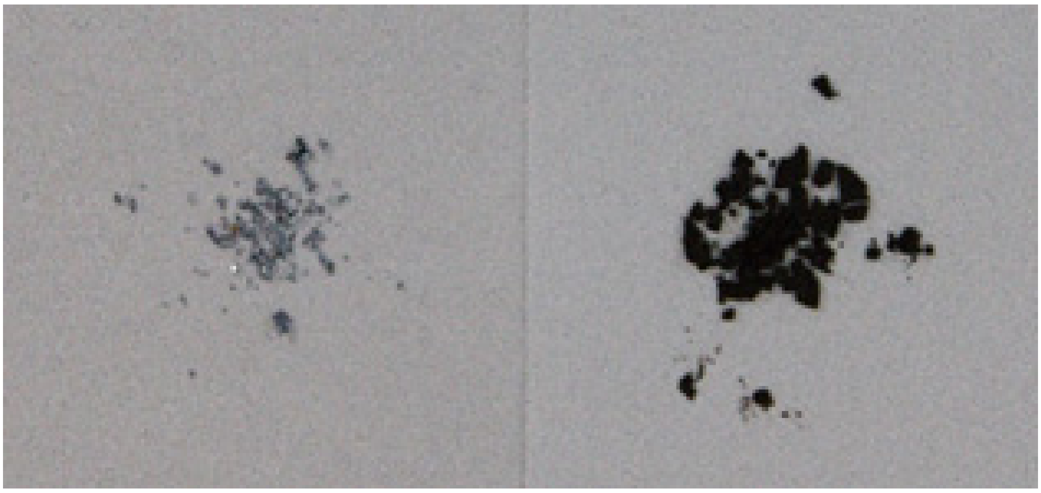

(a) (b)

Figure S1. The appearances of: crystallized metallic osmium **(a)**; osmium powder **(b)**.

$$OsO_4 + Red \rightarrow Os + ox \quad (S1)$$

The extremely high toxicity of osmium tetroxide is the main reason why the various residues containing it must be stabilized and/or recovered.

The present study started from the possibility of extremely easy and fast solubilisation of various residues containing osmium tetroxide in *tert*-butyl alcohol (Figure S2a) and the reduction by bubbling molecular hydrogen to osmium (Figures S2b and S2c).

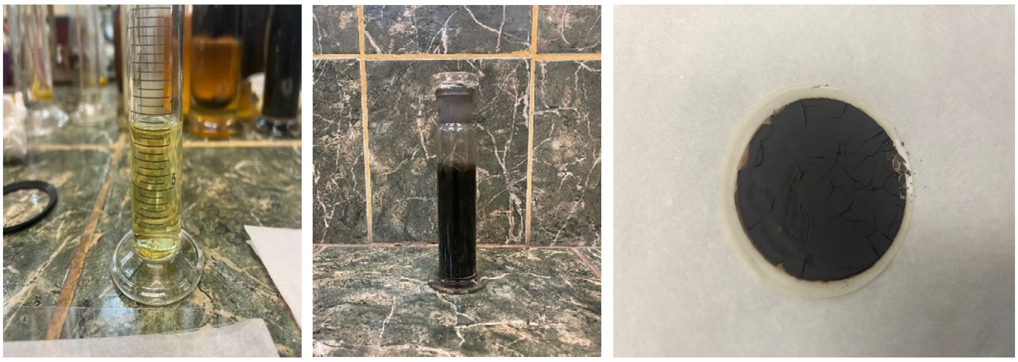

(a) **(b)** **(c)**

Figure S2. Images with: the solution of osmium tetroxide in *tert*-butanol **(a)**; the obtained metallic osmium **(b)**; and the osmium powder retained on a microporous membrane **(c)**.

The reduction reaction is fast and complete after about two hours of bubbling of the molecular hydrogen, but as a precaution the operation lasts four hours. After the bubbling ceases the suspension is filtered on a microporous membrane, and in the filtrate specific color reactions are performed to identify osmium tetroxide. Basically, the described working method leads to a recoverable osmium powder (Figure S3).

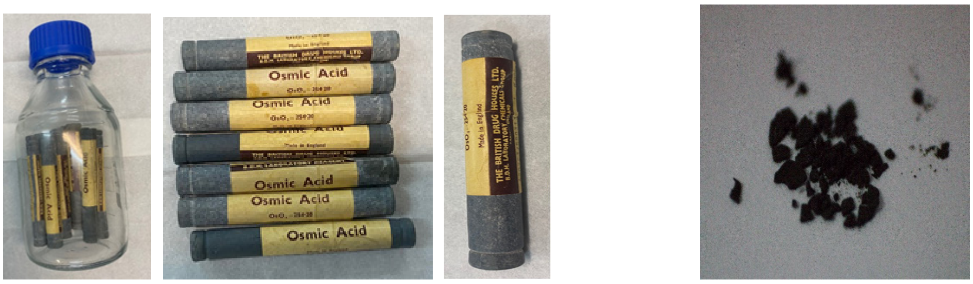

(a) **(b)**

Figure S3. Images with: the bottle in which the osmium tetroxide is kept tightly sealed (expired samples, considered waste) **(a)**; and the osmium powder obtained **(b)**.

Unfortunately, the bulk osmium powder obtained is considered dangerous because it oxidizes over time, emitting osmium tetroxide vapours, is pyrophoric and is difficult to recover from the environments in which it is processed.

The three arguments mentioned above required us to obtain osmium powder on microporous support that has good physical-chemical resistance, but also adequate porosity and pore distribution, namely polypropylene hollow fiber membrane (Figure S4).

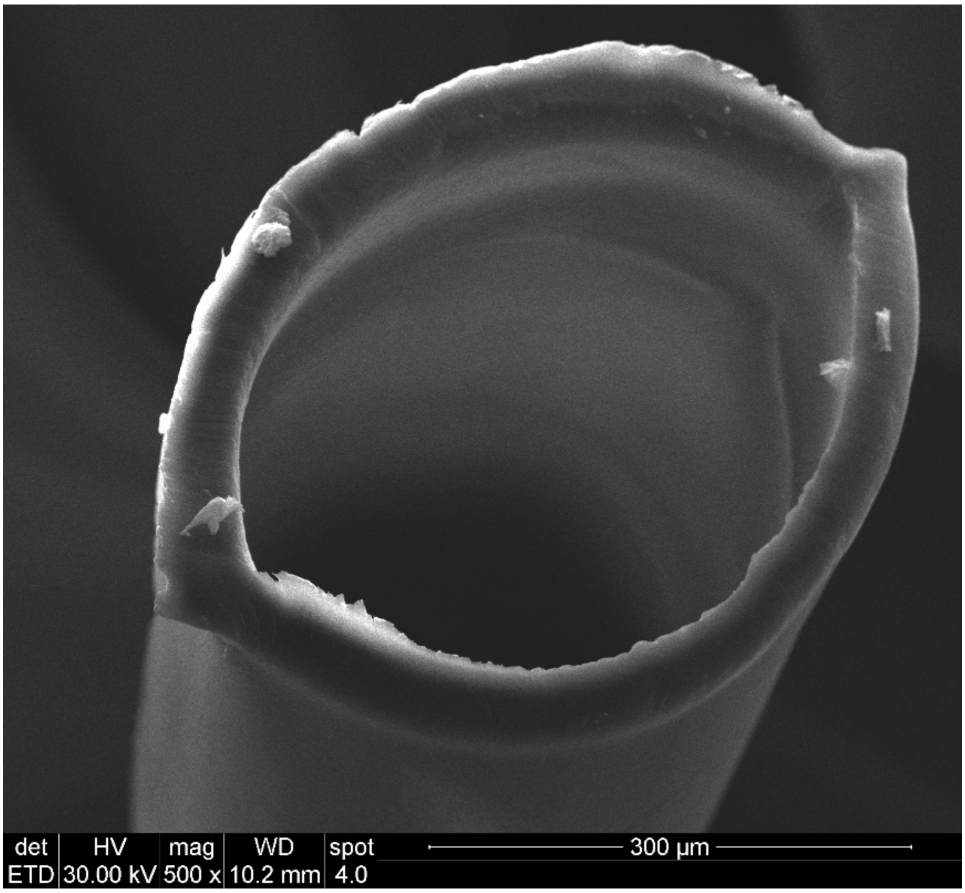
det HV mag WD spot 300 µm
ETD 30.00 kV 500 x 10.2 mm 4.0

(a)

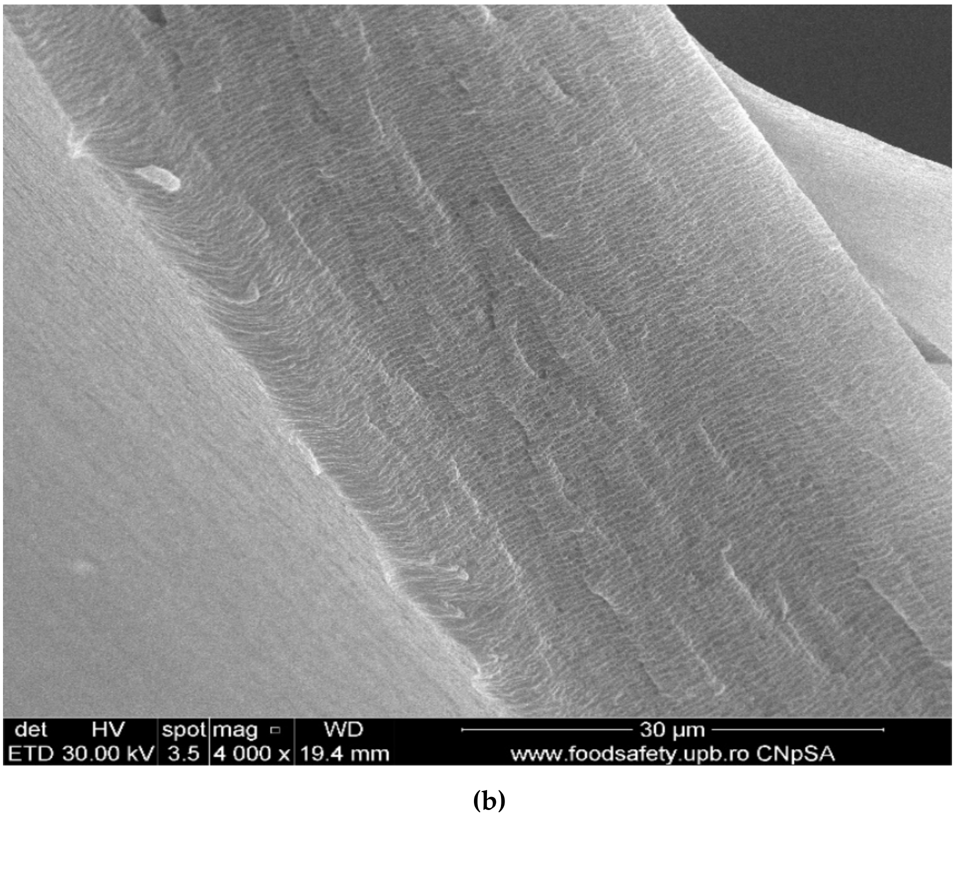
det HV spot mag WD 30 µm
ETD 30.00 kV 3.5 4 000 x 19.4 mm www.foodsafety.upb.ro CNpSA

(b)

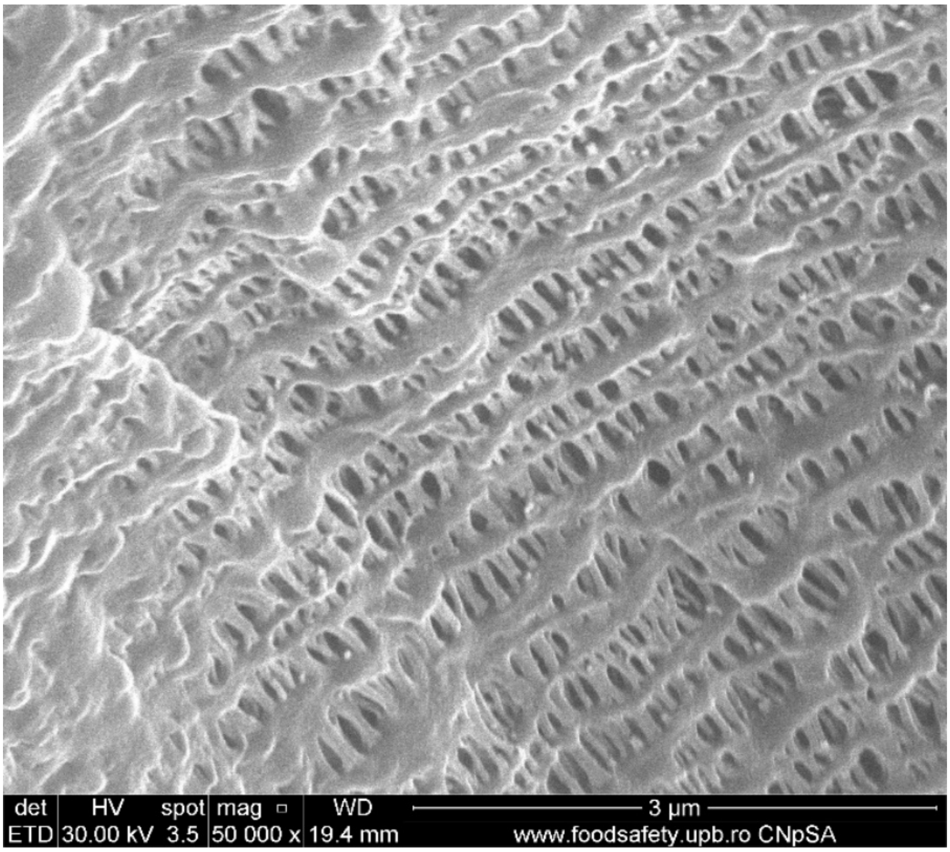

(c)

Figure S4. Scanning electron microscopy (SEM) images of polypropylene hollow fiber membrane support: **(a)** cross-section of the membrane, ×300; **(b)** cross-section of wall membrane, ×4,000; **(c)** pores dimension and distribution details, ×50,000.

The osmium-polypropylene hollow fiber support membrane were obtained in situ. After grinding 10g of dry composite membrane, at a colloidal mill with ceramic balls, metallic osmium was highlighted in the ground powder by XRD analysis (Figure S5a). Of course, the complex organic-inorganic matrix raised problems of interpretation of the obtained data, but the specific lines of metallic osmium were eventually highlighted (Figure S5b).

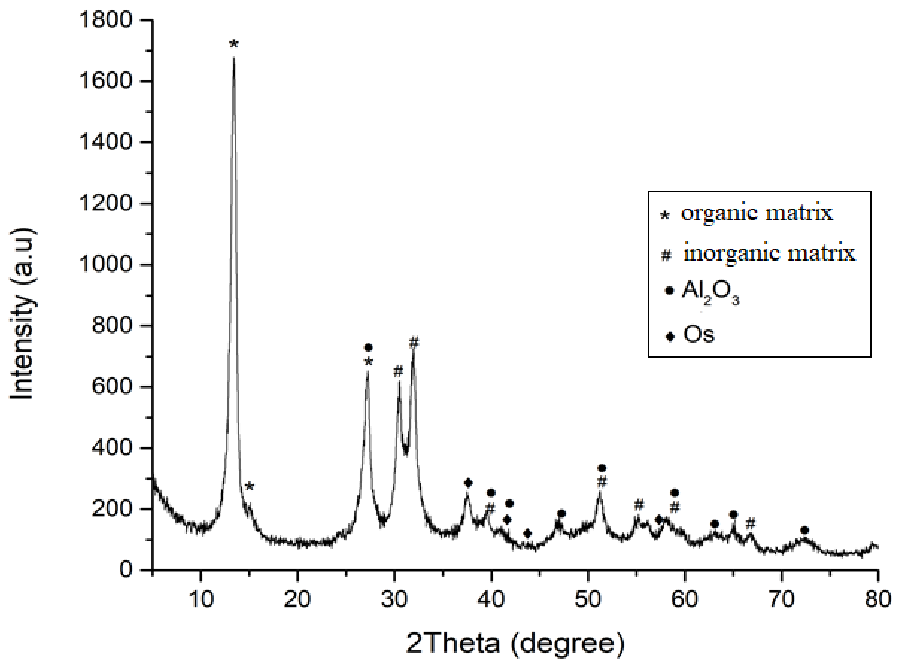

(a)

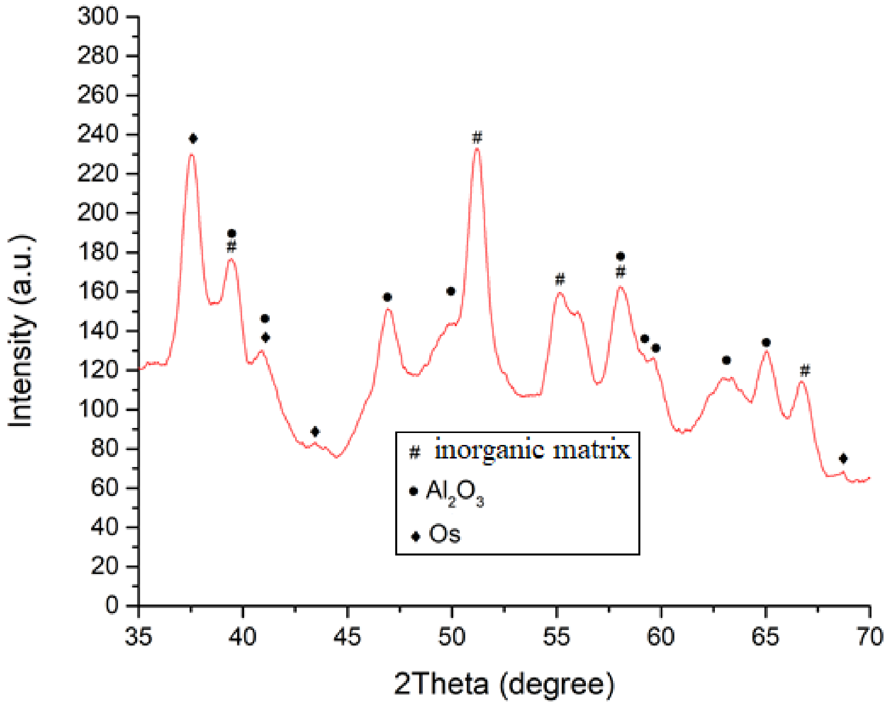

(b)

Figure S5. X-ray diffraction diagrams for polypropylene hollow fiber membrane dust: **(a)** 0–80 theta degree; **(b)** 35–70 theta degree details.

Of course, considering the obtaining of metallic osmium on the surface of polypropylene hollow fiber membrane support, the analysis by scanning electron microscopy (SEM) and energy dispersive spectroscopy analysis (EDAX) are much more appropriate: besides the presence of osmium, its distribution is also revealed (Figure S6). Morphological aspects (SEM) and distribution of osmium and carbon on the membrane surface before (Figure S6a) and after (Figure S6b) processing were highlighted.

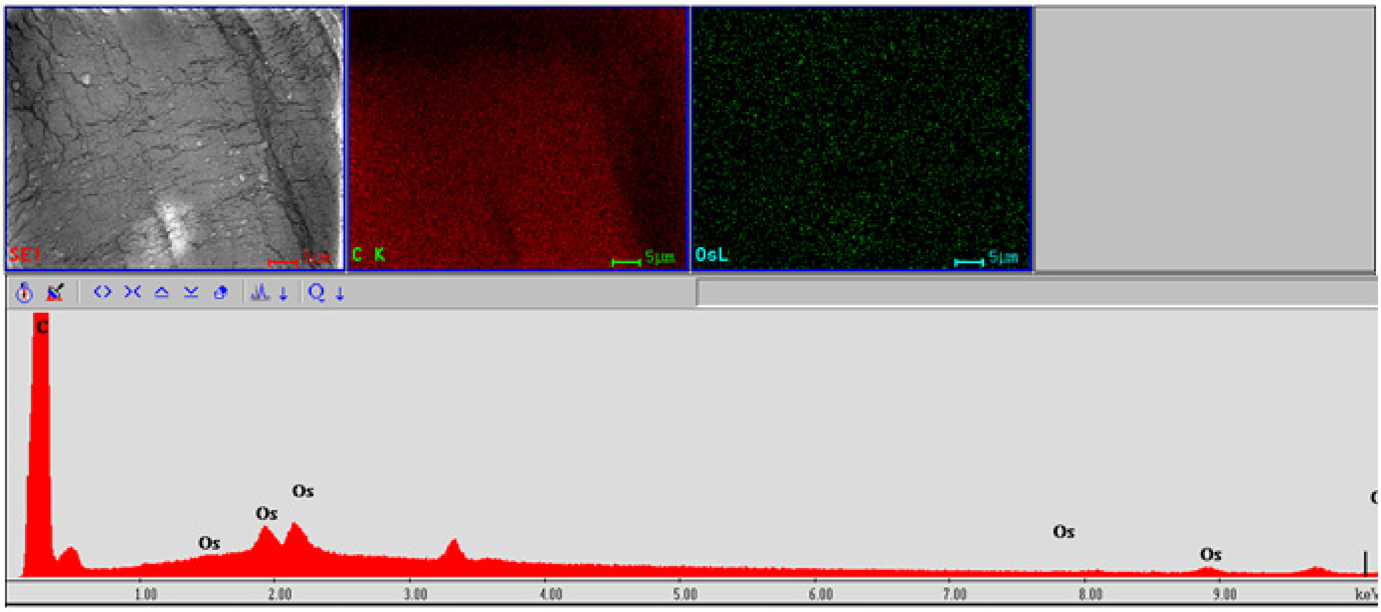

(a)

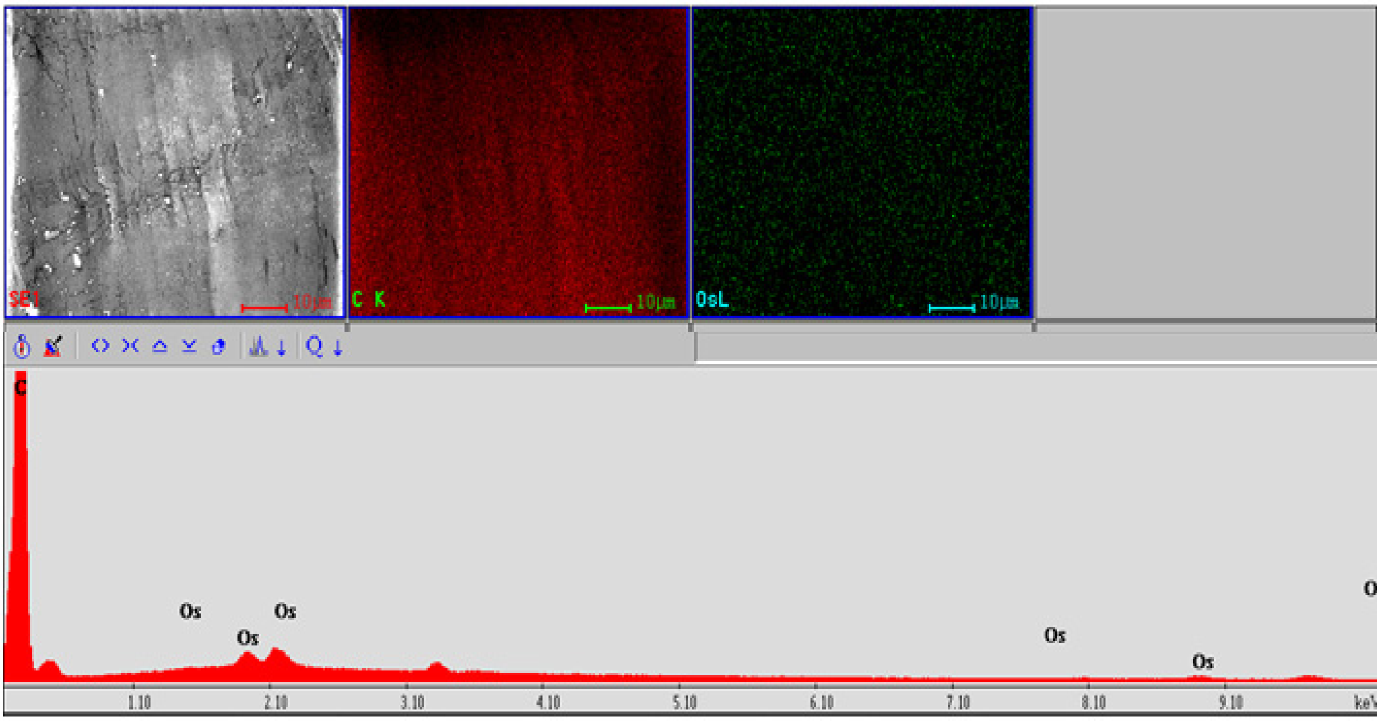

(b)

Figure S6. Scanning electron microscopy (SEM) and energy dispersive spectroscopy analysis (EDAX) on polypropylene hollow fiber membrane support: **(a)** surface of the membrane before processing; **(b)** surface of the membrane after processing.

2. *The composition of the reaction mass to the oxidation with molecular oxygen of 10–undecylenic acid on osmium–polypropylene hollow fiber composite membranes*

Table S1. The composition of the reaction mass to the oxidation with molecular oxygen of 10–undecylenic acid on osmium–polypropylene hollow fiber composite membranes.

**Compounds**	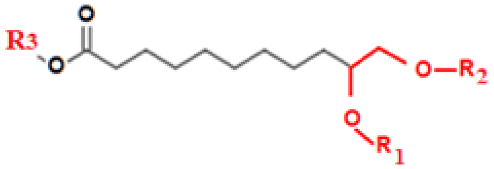				
	R_1, R_2, R_3=H	R_1, R_2, R_3=C_2H_5	R_1, R_2, R_3=C_3H_7	R_1, R_2, R_3=C_4H_9	Others
**Solvent**	**Composition (%)**				
Ethanol	82	16	-	-	2
Propanol	87	-	11	-	2
Tert-Butanol	93	-	-	5	2
Precision (%)	2	5	5	5	5

3. *The specific safety operations on reactional processes and the obtained membranes*

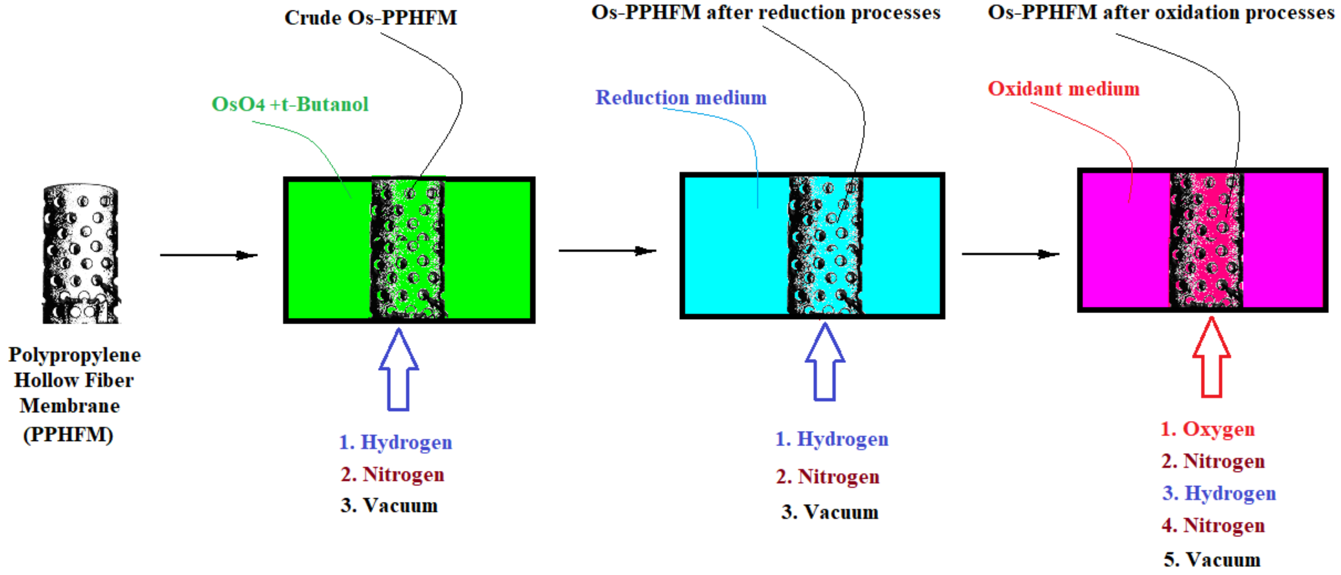

Figure S7. The specific safety operations on reactional processes and the obtained membranes.
